# Do Patches of Flowering Plants Enhance Insect Pollinators in Apple Orchards?

**DOI:** 10.3390/insects14020208

**Published:** 2023-02-19

**Authors:** Myrto Barda, Filitsa Karamaouna, Vaya Kati, Dionysios Perdikis

**Affiliations:** 1 Laboratory of Agricultural Zoology and Entomology, Faculty of Crop Science, Agricultural University of Athens, Iera Odos 75, 11855 Athens, Greece; 2 Scientific Directorate of Pesticides Control and Phytopharmacy, Benaki Phytopathological Institute, 8 Stefanou Delta Str., 14561 Kifissia, Greece; 3 Laboratory of Agronomy, Faculty of Agriculture, Aristotle University of Thessaloniki, 54124 Thessaloniki, Greece

**Keywords:** flowering patches, pollinators, agro-ecosystems, floral resources, weed flora, crop pollination, ecosystem services, groundcover management

## Abstract

**Simple Summary:**

Insect pollinators such as bees contribute to crop production, food security, ecosystem stability and biodiversity in agroecosystems. However, intensification of agricultural practices jeopardizes pollination services in agricultural landscapes mainly through the decline in flower resources for pollinators in farmlands. The study examines a scheme to provide floral resources for insect pollinators in apple orchards potentially contributing to their conservation and enhancing crop pollination. For this reason, flowering mixtures including legume landraces were sown in patches inside apple orchards and compared to the orchard’s weed flora in respect of attraction of pollinators. Pollinators recorded on the sown and wild plant patches were honey bees, wild bees, syrphids and beeflies. The most abundant pollinator of apple was the honey bee but wild bees were also recorded. The sown mixture attracted greater numbers of pollinators and more wild bee taxa compared to the wild plants, but it did not have an effect on pollinators visiting apple flowers. Flowering patches with mixtures of suitable plants in groundcover can enhance pollinator conservation in apple orchards.

**Abstract:**

Apples depend on insect pollination but intensification of agriculture jeopardizes pollination services in agroecosystems. Concerns about the dependency of crop pollination exclusively on honey bees increase the interest in agricultural practices that safeguard wild pollinators in agroecosystems. The purpose of the study was to assess the potential of floral resource provision in apple orchards to enhance the conservation of hymenopterous pollinating insects and potentially the pollination service to the crop. For this reason, flowering plant mixtures sown in patches inside apple orchards were tested against wild plant patches. Pollinator taxa recorded on the sown and wild plant patches were honey bees, wild bees (*Andrena*, *Anthophora*, *Eucera*, *Halictus*, *Lasioglossum*, Megachilidae on both; *Systropha* only on wild plants; *Bombus*, *Hylaeus*, *Sphecodes*, *Nomada*, *Xylocopa* only on sown mixture), syrphids, bee flies. The most abundant pollinator of apple was *A. mellifera* but wild bees were also recorded (*Andrena*, *Anthophora*, *Bombus*, *Xylocopa*, *Lasioglossum*, Megachilidae). The sown mixture attracted a more diverse taxa of pollinators and in greater numbers compared to the weed flora, but it did not have an effect on pollinators visiting apple flowers. Groundcover management with patches of suitable flowering mixtures can enhance pollinator conservation in apple orchards.

## 1. Introduction

Insects play a major role in several fundamental ecological processes of ecosystem functioning and particularly of ecosystem services, including pollination [1,2,3]. Approximately 75% of global food crops and nearly 90% of wild flowering plants are to some degree dependent on animal pollination [4,5], with insects being the most important pollinators in both natural and agricultural settings [6,7]. Pollination of crops is one of the main ecosystem services that affects agriculture [8,9], thus insect pollinators contribute to crop production, food security as well as ecosystem stability and biodiversity in agroecosystems [10,11].

Intensification of agricultural practices jeopardizes pollination services in agricultural landscapes through side effects such as fragmentation or even complete elimination of natural or semi-natural habitats [6,12,13]. Floral resources (pollen, nectar) are vital for pollinators [14,15,16,17], thus the deterioration of flower-rich habitats in farmlands, including hedgerows, species-rich grasslands and legume-rich leys, has been strongly linked with decline in pollinator populations in agroecosystems [18,19,20,21,22,23]. This decline in wild and managed pollinator populations in several regions of the European Union [24] and worldwide [13,25] are detrimental for crop production and biodiversity. Nevertheless, the available literature shows a lack of long-term data on diversity and abundance of bees and crop pollinators, indicating the importance of monitoring them over time [26,27,28,29,30] to enable assessment of declines in pollinating insects both in natural and intensively managed environments and to develop strategies for their support where needed.

In agricultural practice, hives of the domesticated European honey bee (*Apis mellifera* L.) have been utilized during the flowering period of many crops (e.g., almond, apple, cherry, pear, blueberry, melon, mustard) to ensure adequate pollination [31,32,33,34]. However, there are concerns about the dependency of global crop pollination on a single pollinator species [35] as well as on the hive rental costs [36,37]. Moreover, studies have shown that the contribution of wild pollinators (bees other than honey bee) to crop yields may be equal to, or even surpass, that of honey bees [38,39,40,41], hence the growing interest in the development of agricultural practices that safeguard wild pollinators in agroecosystems to ensure the delivery of pollination services [40,42,43,44].

For all the aforementioned reasons, the importance of pollinator conservation in agricultural areas has been acknowledged by the Food and Agriculture Organization and the Institute of European Environmental Policy, which enact policies that comply with the enhancement of ecosystem services, including the International Pollinator Initiative plan of action 2018–2030 [45,46] and the EU Common Agricultural Policy. In fact, approximately 40% of the EU land consists of agricultural production land [47], thus agriculture can contribute significantly to pollinator conservation in the EU. Monitoring of pollinator taxa [48] and conservation measures are being implemented to preserve pollinators in agroecosystems and enhance pollination services [49,50,51,52].

Agri-environmental interventions for insect pollinator conservation typically focus on re-establishing floral resources [53], e.g., restoring species-rich grasslands, sowing field margins with nectar and pollen-rich mixes, and inclusion of flowering species such as legumes in rotations [54,55]. The use of sown flower strips in crops to provide resources for pollinators (mainly Hymenoptera: Apoidea and Diptera: Syrphidae) has been examined by several researchers [8,56,57,58,59,60,61,62,63,64].

Apples, *Malus domestica* Borkh. (Rosaceae), are among the insect pollinated crops as most of their varieties are self-incompatible, and hence they are greatly dependent on insect pollination for high crop yields, fruit set, high fruit quality and economic value [65,66,67,68,69,70]. For this reason, apple growers usually hire hives of *A. mellifera* or *Melipona* spp., add bumblebee (*Bombus terrestris* L.) hives or utilize bee species such as *Osmia* spp. during apple blossom to ensure adequate crop pollination [71,72,73,74]. In addition, studies have shown that wild pollinators, e.g., *Andrena* spp., *Bombus* spp., *Lasioglossum* spp. and Syrphidae, are present in apple orchards [75,76,77,78,79]. However, the need for further research on insect pollinators in apple orchards is emphasized, particularly on the effect of local management practices on apple pollination [80]. Apples are one of the most important fruit crops both globally [81] and in Greece, where they are covering a cultivated area of 9.440 ha with a respective production of 285.9 thousand tons [82].

‘Delicious Pilafa Tripoleos’, hereafter ‘Pilafa’, is a Protected Designation of Origin (PDO) apple landrace, which is produced in all communities of the Prefecture of Arcadia with an altitude higher than 600 m and especially in the region of Tegea, Peloponnese, Greece [83,84]. ‘Pilafa’ apples vary in fruit shape and have a relatively short life of storage [85,86], but they have excellent organoleptic characteristics [87]. Nevertheless, their fruit setting can be limited due to the very short period of flowering and the weather that is usually unstable in Tegea plateau. For this reason, efforts to enhance the attraction of insect pollinators in these orchards can offer a valuable service for the crop sustainability and support the on-farm conservation and valorization of ‘Pilafa’ apples [88,89].

Floral resources for pollinators in perennial crops such as apples are preferably provided in mixtures of plant species of several families such as Apiaceae, Fabaceae, Brassicaceae, etc. [60,62,90]. In this kind of mixtures, the inclusion of traditional varieties would offer a dual benefit since they are part of each country’s cultural heritage, which should be well conserved [91], and their exploitation in agricultural practices would offer an extra incentive to the producer to preserve them on farm. In the present study, two Fabaceae landraces (a landrace of *Vicia faba* L. of Episkopi village in Tegea plateau and a landrace of *Lathyrus sativus* L. of Feneos village in the Peloponnese, Greece) from the region of Tegea were included in a flowering mixture for groundcover in apple orchards. In fact, Fabaceae plants are considered great food resources for the conservation of wild pollinators, such as *Bombus* spp., because of their high-quality pollen and the association of their flower with the bee’s glossa anatomy (e.g., length) [18,92,93,94,95]. Fabaceae pollen is particularly protein-rich, and for this reason nutritious for bee reproduction and larval development [94,96].

The present study tests the hypothesis that sown flowering patches in the groundcover of apple orchards could enhance pollinating insects, mainly pollinating bees, in apple orchards by providing extra flowering resources compared to wild plant flora, also to the benefit of the crop. The aim of the study further extends to contributing to the conservation of insect pollinators in agroecosystems as well as an apple landrace (‘Pilafa’) and two legume landraces (extra flower resources in the groundcover).

## 2. Materials and Methods

### 2.1. Experimental Orchards and Design

The study was carried out in three apple orchards, landrace ‘Pilafa’, located in Tegea plateau, Arcadia, Peloponnese, Greece, during three consecutive growing seasons (2019, 2020, 2021). Two orchards were organic, one palmette (OP) and one goblet (OG) training systems (0.25 ha each) and one IPM (palmette, 0.45 ha) (37.43705, 22.46942; 37.43658, 22.46895; 37.4415, 22.40492). The IPM orchard was located 6 km from the organic orchards while the distance between OG and OP was 100 m (Figure S1). Thus, the study included both temporal and spatial replications, including orchards with different surrounding semi-natural habitats, training system and pest management. Trees in the apple IPM and OG orchards were 14 years old, whereas in the OP orchard, the trees were 5 years old. During the first, baseline year (2019), no intervention was made and the effect of the wild plants/weed flora present in the orchard groundcover on attraction of pollinating insects was determined. The terms wild plants/weed flora will be used interchangeably hereafter, referring to the plant species occurring in the natural, i.e., not sown vegetation. During the next growing seasons (2020, 2021), a mixture of selected annual flowering plant species was established by sowing, to compare with the flowering wild plant species. In each orchard, four strips of the selected flowering mixture and four strips of wild plants were defined (each strip 24 m × 0.80 m) between the tree rows, where plots (patches) for measurements were selected at random from within these strips. The sites of the sown and wild plant strips were located 70–170 m apart (Figure S1).

The experimental design was completely randomized with two treatments (intervention with sown flower mixture and control with wild plants) with six replications (plots of 6.4 m^2^ (8 m × 0.8 m) selected on the strips) per treatment. The plot width (0.80 m) was determined by the wheel axis length of the tractor and other equipment used by the farmers for soil preparation and plant protection applications, which should be able to move freely without compressing the plot, as described in [97].

### 2.2. Establishment of Flower Patches

Nine plant species were selected for the flower mixtures, including 7 broadleaves from 3 families (Apiaceae, Brassicaceae, Fabaceae) and 2 grasses (Poaceae) with an expected combined flowering period extending from March until June (Table 1). The plant species selection was based on criteria such as flower traits that are correlated with pollinator visitation [98], presence of their cultivation in the examined region, exclusion of species that might act as noxious weeds. The grasses, which were wheat landraces, were used (one each year) to investigate their role in facilitating the establishment of ground-dwelling predatory arthropods, which will not be analyzed in the present study, but also in the cohesion of the mixture of flowering plants. There is currently no seed house in Greece that would provide seeds of indigenous plant species in large quantities. Therefore, commercial varieties and landraces of Tegea region (*V. faba* ‘Episkopi’, *L. sativus* ‘Fava Feneou’ PGI) were utilized.

A mixture was preferred over single plant species to ensure longer periods of flowering and to provide greater diversity of suitable flowers for both honey bees and wild bees, thus avoiding behavioral competition in foraging between them over a single species [99]. The composition of the mixture was optimized in the third year (2021) with the appropriate replacement or exclusion of certain plant species based on their establishment and general performance (flowering period and duration, attractiveness to pollinators). General criteria in the mixtures’ composition included relative species growth and flowering characteristics, but also the cost and availability of the seed material. The seed weight for each species in the mixture (Ws) was calculated according to the equation by [63]:

Ws = Ps × (1/ESR) × (1/Pg) × Tp × A × TGW/1000, where Ps = Target percentage of plants per species (see Table 1); ESR = Estimated survival rate of germinated seeds (ranging between 50 and 70% depending on the plant species); Pg = Seed germination percentage (petri dish assays); Tp = Total target number of plants/m^2^; A = Area to be sown (m^2^); TGW = Thousand grain weight (g).

A rotary tiller was used for soil preparation prior to sowing of the mixtures in the flowering plots while the control plots were not tilled. Broadcast sowing of the mixtures was performed by hand in autumn (November 2020, 2021). Seed volume was augmented with a bulking material (river sand) to facilitate a uniform sowing. Seeds were covered with shallow raking and the soil surface was rolled to ensure good seed/soil contact. No irrigation was required since the soil moisture was adequate for seed germination and seedling establishment. It should be mentioned that the growers followed the usual agricultural practice regarding weed management in the control plots. There was no application of herbicides in the sown or wild plant patches in any of the orchards; instead, farmers managed the weeds with one to two rotary tillers, from March to June, without affecting the sown mixture patches.

### 2.3. Landscape Elements around the Experimental Orchards

Landscape elements, such as semi-natural habitats, around the experimental orchards were recorded as such areas are a key factor for bee conservation [100,101,102]. Therefore, habitats (including semi-natural) in the proximity (at the border line) of the experimental apple orchards were recorded and are presented below. The annual plant species mentioned were in bloom after apple flowering (mid-May).

Neighboring habitats to the organic palmette apple (OP) orchard. These habitats included: (a) uncultivated area with *Cardaria draba* (L.) Desv. (Brassicaceae), *Vicia sativa* L. (Fabaceae), *Galium aparine* L. (Rubiaceae), *Euphorbia* sp. (Euphorbiaceae), *Scabiosa* sp. (Caprifoliaceae), *Medicago polymorpha* L. (Fabaceae), *Cirsium* sp. (Asteraceae), Brassicaceae (e.g., *Sinapi*s sp., *Raphanus* sp.), *Daucus carota* L. (Apiaceae), *Ranunculus* sp. (Ranunculaceae), *Echium* sp. (Boraginaceae); (b) uncultivated area mainly with *Trifolium repens* L. (Fabaceae) and *Avena sterilis* L. (Poaceae), and small numbers of *Lamium amplexicaule* L. (Lamiaceae), *C. draba*, *Euphorbia* sp., *Ranunculus* sp., Brasicaceae and *V. sativa*; (c) a cultivated cherry orchard; (d) an abandoned old cherry orchard; (e) natural hedges of wild *Prunus avium* L. (Rosaceae) and *Rubus* sp. (Rosaceae); (f) hedge-field margin of *Rubus* sp., Poaceae (e.g., *A. sterilis*), *L. amplexicaule*, *Vicia villosa* Roth. (Fabaceae), and *Scabiosa* sp.

Neighboring habitats of the organic goblet apple (OG) orchard. These semi-natural habitats included: (a) a cherry orchard and groundcover mainly with *T. repens* and *M. polymorpha*; (b) forest trees of hawthorns, walnuts wild sour cherries, brambles, wild roses and annual plants of *V. villosa*, *Scabiosa* sp., *T. repens*, *Anthemis* sp. (Asteraceae), *Vinca* sp. (Apocynaceae), *Papaver* sp. (Papaveraceae), *D. carota*, *A. sterilis*, *Cirsium* sp., *M. polymorpha*; (c) an abandoned old cherry orchard.

Neighboring habitats of the IPM apple orchard. These habitats included: (a) an apple orchard and groundcover mainly with *Matricaria chamomilla* L. (Asteraceae); (b) a cherry orchard; (c) a cultivated area with cabbage, cauliflower and other vegetables such as peppers, eggplants, rocket, potatoes and plants such as *M. chamomilla*, *Capsella bursa-pastoris* L. (Brassicaceae), *Veronica persica* Poir. (Plantaginaceae), *Ranunculus* sp.

### 2.4. Flowering and Insect Presence Measurements

Measurements included recording of plant and flower cover and attracted insect pollinators. The measurements were performed during the flowering period of the sown plants (from April to June), the wild plants in the control plots (from April to June), and the apple trees (mid to late April). The methodology was as described before [62,63,64] adapted for the experimental design of the current study. More specifically, the plant cover (total) and flower cover (total and per plant species) were visually estimated and expressed as percentage cover of the plot area (6.4 m^2^), in all examined plots (sown and control (wild plants)). Plant species were identified in situ or, when necessary, in the laboratory using the botanical identification key Flora Europaea [103]. Visits of insect-pollinators (with a focus on bees, wild bees, syrphid flies and bee flies) at the flowers of the sown mixture, the wild plants as well as the apple blossom in trees adjacent to sown and control plots were recorded by visual observation, for 1 min per plot (6.4 m^2^ or 3 trees) (hereafter visitation will refer to mean number of visits/6.4 m^2^/min). The measurements of pollinator visitation on apple blossoms were taken on flowers of three consecutive trees of a row located between sown or wild plant patches. In the palmette orchards, the one-minute visual observation on apple flowers was conducted on the vertical leaf wall area of the trees. In the goblet orchard, each tree was perceived as a cube and the measurements were taken on the surface area that was in the same cardinal point as the examined groundcover plot. All counts were conducted between 10:00 and 16:00 h at temperatures in the range of 11 to 33 °C and no more than half the sky was covered with clouds, when possible, e.g., in year 2019 the weather was cloudy (more than half the sky covered with clouds) during the period (10 days) of apple flowering. Wind velocity was also recorded to ensure that it did not exceed 4.35 m/s. The pollinators that could not be identified in genus level in situ were collected by sweeping net sampling, stored in the freezer (−18 °C) and identified by the first author in generic level (or species level when possible) under a stereo-microscope (OLYMPUS SZ61, U.K) using taxonomic keys [16,104].

During the baseline year (spring 2019), plant cover and flower cover of wild plants as well as pollinator visits on flowers of wild plants and apple trees were recorded. These measurements were conducted at one site with six plots in each organic orchard, and at two sites (IPMa, IPMb—six plots each) in the IPM orchard due to its larger total area and variability in plant species across the groundcover area. The baseline recordings were deemed necessary in order to assess the effect of the intervention in the following years.

### 2.5. Statistical Analysis

The effect of groundcover (sown flowering mixture/wild plants), recording date and their interaction on the visitation of honey bees, wild bees in the flower patches and apple blossom were determined using 2-way ANOVA (α = 0.05) for each orchard (IPM, OP, OG) and for each experimental year separately. The visitation of Syrphidae and Bombyliidae was too low to allow a statistical analysis. When the effect of both factors and their interaction was significant or either factor and the interaction was significant, the means for the respective factors were separated using Tukey’s HSD test (α = 0.05). The statistical analyses were performed using the statistical package JMP [105].

## 3. Results

### 3.1. Plant Mixture Establishment and Flowering

**Figure 1:**
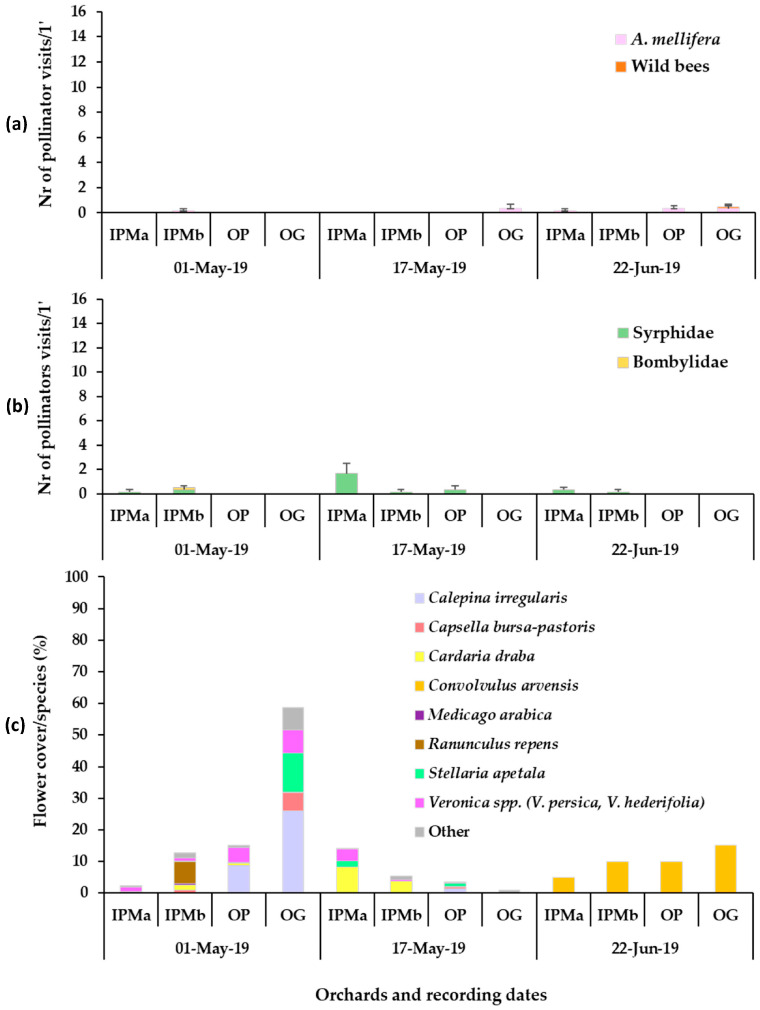
Mean numbers of (**a**) Hymenoptera (honey bees, wild bees), (**b**) Diptera pollinator visits recorded on groundcover patches of natural vegetation (wild plants) (1 min/6.4 m^2^) and (**c**) mean flower cover percentage per plant species in the patches in apple orchards in the baseline year, 2019. IPMa: IPM field site a, IPMb: IPM field site b, OP: organic palmette field, OG: organic goblet field. Vertical bars represent standard error of means. Other species: *Senecio vulgaris*, *Taraxacum officinale*, *Cardamine hirsute*, *Raphanus raphanistrum*, *Cerastium glomeratum*, *Euphorbia* sp., *Medicago polymorpha*, *Trifolium repens*, *Geranium disectum*, *Alopecurus myosuroides*, *Poa annua*, *Galium aparinae.*

**Figure 2:**
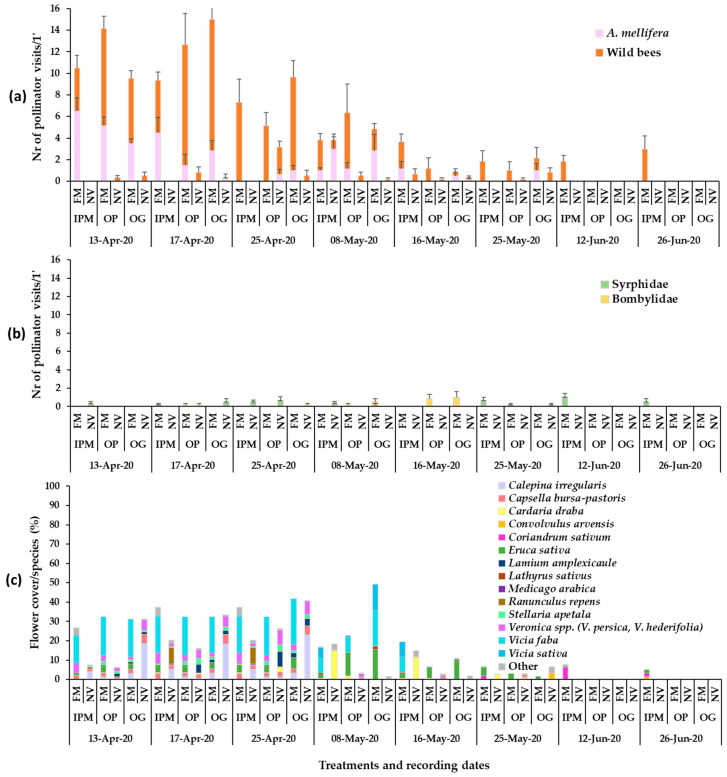
Mean numbers of (**a**) Hymenoptera pollinator visits (honey bees, wild bees) and (**b**) Diptera visits on ground cover patches (1 min/6.4 m^2^) in apple orchards and (**c**) mean flower cover percentage per plant species in the patches in year 2020. FM: sown flowering mixture; NV: natural vegetation (wild plants); IPM: IPM orchard, OP: organic palmette orchard, OG: organic goblet orchard. Vertical bars represent standard error of means. Other species: *Brassica nigra*, *Cardamine hirsuta*, *Geranium disectum*, *Malva* spp., *Matricaria chamomila*, *Medicago polymorpha*, *Poa annua*, *Raphanus* sp., *Senecio vulgaris*, *Sonchus oleraceus*, *Taraxacum officinale*, *Trifolium repens*, *Cerastium glomeratum*, *Draba muralis*, *Euphorbia* sp., *Galium aparinae*, *Lythospermum arvense*.

**Figure 3:**
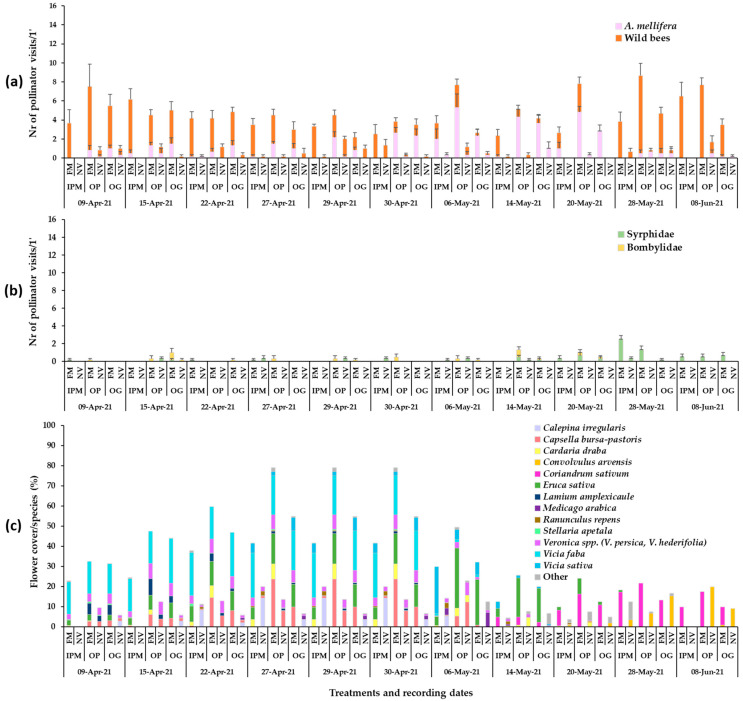
Mean numbers of (**a**) Hymenoptera, (**b**) Diptera pollinator visits (honey bees, wild bees) and Diptera visits on ground cover patches (1 min/6.4 m^2^) in apple orchards and (**c**) mean flower cover percentage per plant species in the patches in year 2021. FM: sown flowering mixture; NV: natural vegetation (wild plants); IPM: IPM orchard, OP: organic palmette orchard, OG: organic goblet orchard. Vertical bars represent standard error of means. Other species: *Brassica nigra*, *Cardamine hirsuta*, *Geranium disectum*, *Malva* spp., *Matricaria chamomila*, *Medicago polymorpha*, *Poa annua*, *Raphanus* sp., *Senecio vulgaris*, *Sonchus oleraceus*, *Taraxacum officinale*, *Trifolium repens*, *Alopecurus myosuroides*, *Campanula* sp., *Chrysanthemum* sp., *Fumaria officinalis*, *Papaver rhoeas*, *Picris hieracioides*, *Vicia incisa*.

The flowering wild plant species recorded in each apple orchard during the baseline year (2019) were mainly *Veronica* spp., *C. bursa-pastoris*, *Calepina irregularis* (Asso) Thell. (Brassicaceae), *Stellaria apetala* Ucria (Caryophyllaceae) and *Ranunculus repens* L. (Ranunculaceae) in early May, *C. draba* in mid-May, and *Convolvulus arvensis* L. (Convolvulaceae) in late June (Figure 1). Plant cover in the baseline year ranged between 60 and 100%. In year 2020, in the patches of sown mixtures, the broadleaved species that germinated and reached flowering were *V. sativa*, *V. faba*, *Eruca sativa* Mill. (Brassicaceae). *Anethum graveolens* L. (Apiaceae) germinated in small numbers but did not reach flowering and *L. sativus* reached flowering in small numbers, while *Coriandrum sativum* L. (Apiaceae) reached flowering only in the IPM orchard. *Lathyrus sativus*, which is cultivated as an annual spring crop in the region of Tegea plateau but was selected to participate in the sown mixture as a winter crop in our effort to utilize landraces in the sown mixture, did not establish well. Therefore, in year 2021, *L. sativus* was excluded from the mixture. In year 2021, *V. sativa*, *V. faba*, *E. sativa* and *C. sativum* germinated and reached flowering in all experimental orchards. *Anethum graveolens* and *Foeniculum vulgare* Mill. (Apiaceae) did not reach flowering. *Triticum aestivum* L. (Poaceae) germinated and fulfilled its cohesion role in the patches of the sown mixture in both years. The broadleaved sown plant species reached flowering in the successive order: *V. faba* > *E. sativa* > *V. sativa* > *L. sativus* > *C. sativum*. The duration of the flowering in the plant mixtures was from late March/early April to June/July depending on the experimental year. The plant species in the weed patches and the sown mixtures which reached the flowering stage in each orchard are presented in Figure 1, Figure 2 and Figure 3. These mainly included *V. persica*, *Veronica hederifolia* L. (Plantaginaceae), *C. bursa-pastoris*, *C. irregularis*, *S. apetala*, *C. draba*, *L. amplexicaule*, *C. arvensis*. Plant cover in the sown flower patches ranged between 70 and 100% and in the wild plant patches between 40 and 100%; flower cover is shown in Figure 1, Figure 2 and Figure 3. The apple trees were in bloom from mid- to late April.

### 3.2. Presence of Pollinators in Sown/Wild Plant Patches and on Apple Blossom

In the baseline year (2019), low visitation (mean number of visits/6.4 m^2^/min) of honey bees was recorded on the wild plant patches in all orchards. In fact, the only wild bee recorded that year appeared in the OG orchard, where one visit of *Systropha curvicornis* Scopoli (Halictidae) was recorded on *C. arvensis* in late June. No statistical differences were indicated between the recording dates in all orchards (Table S1, Figure 1).

In the first year of groundcover management with the selected flowering mixture (2020), the groundcover, the recording date, and their interaction had a significant effect on the mean number of both honey bee and wild bee visits in all orchards (two-way ANOVA; **Honey bees:** IPM: Groundcover: F_1,80_ = 19.40, *p* < 0.0001; Date: F_7,80_ = 10.12, *p* < 0.0001; Groundcover*Date: F_7,80_ = 11.78, *p* < 0.0001; OP: Groundcover: F_1,70_ = 25.46, *p* < 0.0001; Date: F_6,70_ = 11.81, *p* < 0.0001; Groundcover*Date: F_6,70_ = 13.54, *p* < 0.0001; OG: Groundcover: F_1,70_ = 20.52, *p* < 0.0001; Date: F_6,70_ = 3.30, *p* < 0.0001; Groundcover*Date: F_6,70_ = 4.74, *p* < 0.0001; **Wild bees:** IPM: Groundcover: F_1,80_ = 66.59, *p* < 0.0001; Date: F_7,80_ = 2.38, *p* = 0.0285; Groundcover*Date: F_7,80_ = 2.93, *p* = 0.0089; OP: Groundcover: F_1,70_ = 38.62, *p* < 0.0001; Date: F_6,70_ = 7.25, *p* < 0.0001; Groundcover*Date: F_6,70_ = 5.63, *p* < 0.0001; OG: Groundcover: F_1,70_ = 51.82, *p* < 0.0001; Date: F_6,70_ = 10.58, *p* < 0.0001; Groundcover*Date: F_6,70_ = 11.23, *p* < 0.0001). For this reason, the results for the mean number of honey bee and wild bee visits were examined separately for each recording date.

The mean numbers of honey bee and wild bee visits on the sown flowering mixture and the wild plant patches in 2020 are shown in Figure 2. The flowering sown patches in the apple orchards attracted primarily wild bees and fewer honey bees. In the first recording date (13 April), mean honey bee visits on the flowering mixture were significantly higher compared to the visits on the wild plant patches in all orchards. In the second recording date (17 April), a significant difference between the flowering mixture and the wild plant patches was evident in IPM and OG orchards, whereas in the third recording date (25 April), a significant difference was recorded only in the OG orchard. In the following five recording dates (early May to late June), no significant differences were recorded in honey bee visits between the sown and wild plant patches (Tukey’s HSD test, *a* = 0.05 for each orchard/date; Supplementary Table S1). Honey bee visitation in the sown patches was higher compared to the wild plant patches in mid-April when in all orchards the main flowering species was *V. faba (E. sativa*, *Veronica* spp., *C. bursa-pastoris* also present), Figure 2. In the IPM orchard, higher visitation of honeybees in wild plant patches was recorded in early May when the main flowering species was *C. draba* and in the OG orchard when the main flower cover was from *C. arvensis* (Tukey’s HSD test, *a* = 0.05 for each orchard/groundcover; Supplementary Table S1).

Regarding wild bees, patches with the flowering mixture attracted a significantly higher number of visits compared to those with the wild plants. This was evident in all orchards just before full blooming of apple trees (13 April) and continued throughout apple blossom (17 April, 25 April). In later measurements, after the crop flowering (8 May), this was also evident in the IPM and the OG orchards; the main flowering species in the sown patches during all that three-week period were *V. faba*, *V. sativa* and *E. sativa*, the two latter species at the end of the period (Tukey’s HSD test, *a* = 0.05, for each orchard/date; Supplementary Table S1). In addition, significantly higher wild bee visits were recorded in the flowering mixture of the IPM orchard in June (12 June, 26 June) on flowers of *C. sativum* (coriander, the only flowering species in that period, failed to establish in the organic orchards) (Tukey’s HSD test, *a* = 0.05, for each orchard/groundcover; Supplementary Table S1).

In 2021, the groundcover type, the recording date and their interaction had a significant effect on the mean number of honey bee visits in the patches in both organic orchards, whereas in the IPM orchard, no significant differences were recorded in honey bee visitation between the flowering mixture and the wild plant patches (two-way ANOVA; **Honey bees:** IPM: Groundcover: F_1,110_ = 7.04, *p* = 0.0091; Date: F_10,110_ = 13.17, *p* = 0.0013; Groundcover*Date: F_10,110_ = 8.90, *p* = 0.055; OP: Groundcover: F_1,110_ = 91.22, *p* < 0.0001; Date: F_10,110_ = 7.37, *p* < 0.0001; Groundcover*Date: F_10,110_ = 8.16, *p* < 0.0001; OG: Groundcover: F_1,110_ = 49.35, *p* = 0.0004; Date: F_10,110_ = 3.59, *p* < 0.0001; Groundcover*Date: F_10,110_ = 2.32, *p* = 0.0161). Overall, the sown patches in the organic orchards had a higher visitation of honey bees compared to the wild plant patches from mid- / late April to late May. In the OP orchard, higher bee visitation was recorded in sown patches from the end of April (main flower cover from *V. faba* = *C. bursa pastoris = E. sativa > Veronica* sp. = *C. draba*) until 20 May (main flower cover from *E. sativa* and *E. sativa + C. sativum* at the end of this period) (Figure 3; Tukey’s HSD test, *a* = 0.05, for each groundcover/date; Supplementary Table S1).

The effect of groundcover management, recording date and their interaction was significant also on the wild bee visitation in the patches (two-way ANOVA; **Wild bees:** IPM: Groundcover: F_1,110_ = 122.74, *p* < 0.0001; Date: F_10,110_ = 2.38, *p* = 0.0135; Groundcover*Date: F_10,110_ = 2.95, *p* = 0.0025; OP: Groundcover: F_1,110_ = 101.27, *p* < 0.0001; Date: F_10,110_ = 6.39, *p* < 0.0001; Groundcover*Date: F_10,110_ = 6.06, *p* < 0.0001; OG: Groundcover: F_1,110_ = 79.85, *p* < 0.0001; Date: F_10,110_ = 6.09, *p* < 0.0001; Groundcover*Date: F_10,110_ = 5.01, *p* < 0.0001). Higher wild bee visitation was recorded on the sown patches compared to the wild plant patches throughout the observation period in the IPM orchard (mixed flower cover), while in the organic orchards, higher visitation of wild bees was observed during the middle weeks of April (mixed flower cover) and the end of May–early June (mainly coriander flowering) (Figure 3; Tukey’s HSD test, *a* = 0.05, Supplementary Table S1).

During the visual observations, a small number of visits of Diptera pollinators of Syrphidae and Bombyliidae was also recorded on the flowers of the sown mixture and wild flora (Figure 1b, Figure 2b and Figure 3b). *Eruca sativa* and *C. sativum* were the main plants to attract Diptera pollinators in the sown flowering mixture patches, while *Veronica* spp., *C. bursa-pastoris*, *C. irregularis*, *C. draba* and *C. arvensis* were the main plants to attract syrphids and bee flies in the wild plant patches.

The apple landrace ‘Pilafa’ has a very short period of flowering, approximately 10 to 15 days, usually mid- to late April, depending on the weather conditions. At that time of the season, the weather is usually unstable in Tegea plateau affecting the bee presence on the apple blossoms (Figure S2).

**Figure 4:**
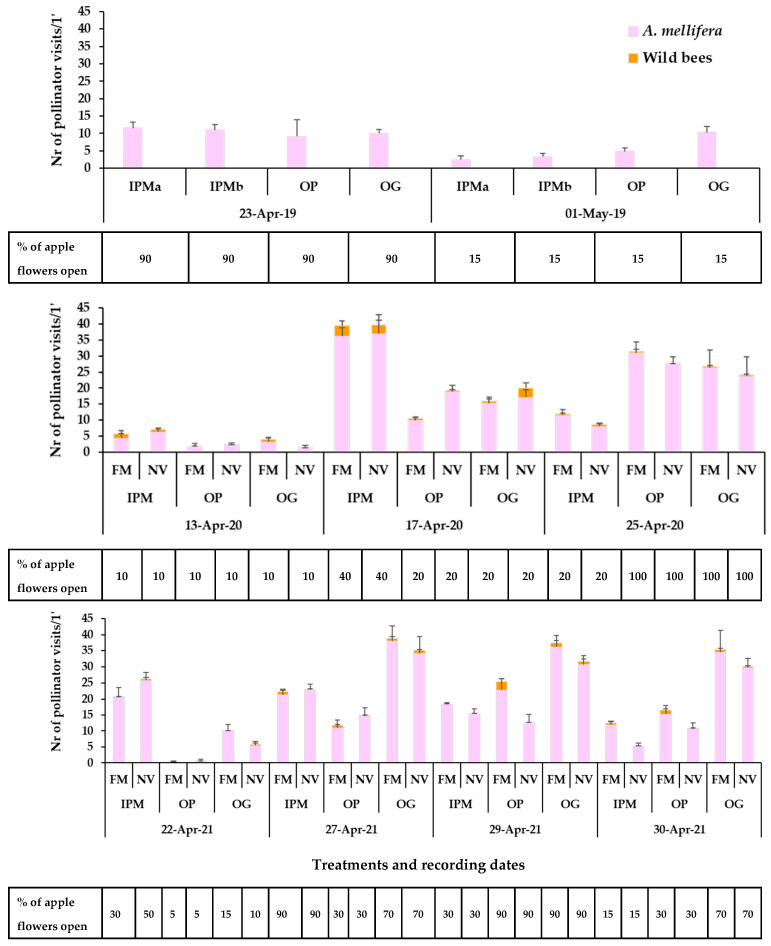
Mean numbers of Hymenoptera pollinator visits (honey bees, wild bees) on apple flowers recorded for 1 min/6.4 m^2^ in the baseline year 2019, 2020, 2021. The horizontal bar below each graph represents the percentage of apple flowers open in each plot. IPM: IPM orchard with site (a) and (b) for the baseline year, OP: organic palmette orchard, OG: organic goblet orchard, FM: sown flowering mixture; NV: natural vegetation (wild plants). Vertical bars represent standard error of means.

The honey bee visits on apple flowers (e.g., range of means in full bloom: 9–11 visits/1 min/plot in the three orchards) in the baseline year (2019) did not differ between the fields (two-way ANOVA; Honey bees: Orchard: F_3,40_ = 1.06, *p* = 0.3763; Date: F_1,40_ = 11.67, *p* = 0.0015; Orchard*Date: F_3,40_ = 1.89, *p* = 0.1465). No wild bees were recorded on apple blossoms in any of the orchards (Figure 4). In the experimental years with sown patches in the orchard, both honey bee visits (e.g., range of means in full bloom in the three orchards: year 2020/ IPM: 36- 37, OP: 28–31, OG: 24–26 visits/1 min/plot; year 2021/ IPM: 21–23, OP: 13–23, OG: 31–36 visits/1 min/plot) and few wild bee visits (e.g., range of means in full bloom in the three orchards: year 2020/ IPM: 2.66–3.33, OP: 0.33–0.67, OG: 0.67–2.83 visits/1 min/plot; year 2021/ IPM: 0.17 – 1.00, OP: 0.00–2.67, OG: 1.00–1.17 visits/1 min/ plot) were recorded on apple flowers of trees adjacent to the sown flowering patches and to the wild plant patches. Overall, the groundcover did not have a predominant significant effect on honey bee visits on apple flowers while the effect of ‘recording date’ was significant in all orchards indicating the role of the abundance of apple flowers in the attraction of *A. mellifera*.

More specifically, regarding the honey bee visitation on the apple blossom, groundcover did not have a significant effect, while the effect of recording date was significant as well as, in some cases, their interaction (two-way ANOVA; **Year 2020:** IPM: Groundcover: F_1,30_ = 0.009, *p* = 0.9248; Date: F_2,30_ = 69.99, *p* < 0.0001; Groundcover*Date: F_2,30_ = 0.51, *p* = 0.6034; OP: Groundcover: F_1,30_ = 1.68, *p*= 0.2042; Date: F_2,30_ = 99.02, *p* < 0.0001; Groundcover*Date: F_2,30_ = 5.29, *p* = 0.0107; OG: Groundcover: F_1,30_ = 0.09, *p* = 0.7669; Date: F_2,30_ = 19.87, *p* < 0.0001; Groundcover*Date: F_2,30_ = 0.21, *p* = 0.8068; **Year 2021**: IPM: Groundcover: F_1,50_ = 0.38, *p* = 0.5382; Date: F_4,50_ = 69.73, *p* < 0.0001; Groundcover*Date: F_4,50_ = 3.93, *p* = 0.0074; OP: Groundcover: F_1,50_ = 2.75, *p* = 0.1029; Date: F_4,50_ = 31.04, *p* < 0.0001; Groundcover*Date: F_4,50_ = 3.51, *p* = 0.0132; OG: Groundcover: F_1,50_ = 2.69, *p* = 0.1068; Date: F_4,50_ = 42.38, *p* < 0.0001; Groundcover*Date: F_4,50_ = 0.18, *p* = 0.9467). Looking at the cases of significant groundcover × recording date interaction, in spring 2020, honey bee visits in the OP orchard were significantly higher on the apple blossom of the trees adjacent to the wild plant patches on 17 April (Tukey’s HSD test, *a* = 0.05, Supplementary Table S2). Ιn spring 2021, honey bee visitation in OP and IPM orchards was higher on apple blossom of the trees next to the sown flowering patches at the end of April (Tukey’s HSD test, *a* = 0.05, Supplementary Table S2).

Concerning wild bees, the effect of groundcover was not significant on wild bee visitation on apple blossom except for the OP orchard in 2021 at the end of April, when a higher number of wild bee visits was recorded on flowers of trees adjacent to the sown flowering patches compared to wild plant patches (two-way ANOVA; **Year 2020:** IPM: Groundcover: F_1,30_ = 0.21, *p* = 0.6481; Date: F_2,30_ = 3.43, *p* = 0.0452; Groundcover*Date: F_2,30_ = 0.10, *p* = 0.8976; OP: Groundcover: F_1,30_ = 1.14, *p* = 0.2928; Date: F_2,30_ = 1.23, *p* = 0.3042; Groundcover*Date: F_2,30_ = 0.32, *p* = 0.7278; OG: Groundcover: F_1,30_ = 0.67, *p* = 0.4182; Date: F_2,30_ = 2.40, *p* = 0.1075; Groundcover*Date: F_2,30_ = 1.97, *p* = 0.1568. (**Year 2021:** OP: Groundcover: F_1,50_ = 20.69, *p* = 0.0001; Date: F_4,50_ = 7.31, *p* = 0.0001; Groundcover*Date: F_4,50_ = 7.28, *p* = 0.0001; Tukey’s HSD test, *a* = 0.05, Supplementary Table S2.)

### 3.3. Bee Fauna Composition and Other Main Fauna of Flower Visitors in Association to Flower Species

**Figure 5:**
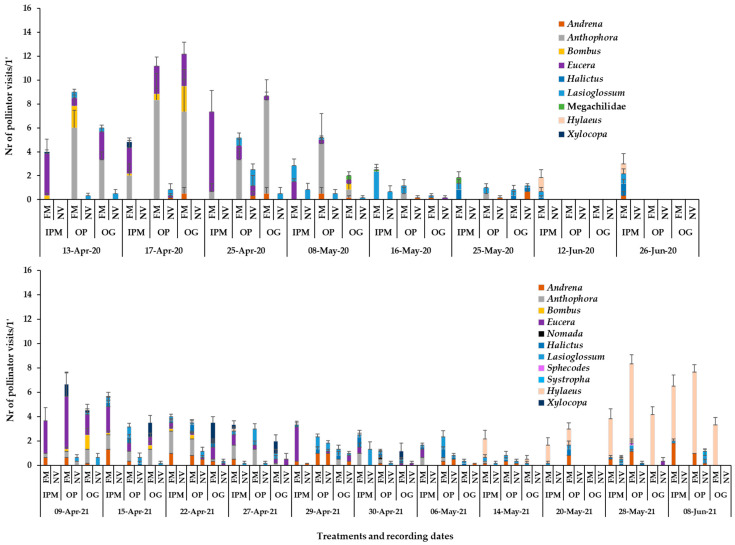
Mean numbers of wild bees recorded in groundcover patches (1 min/6.4 m^2^) in apple orchards in 2020 and 2021. Wild bee taxa are indicated by different colour in each column. FM: sown flowering mixture; NV: natural vegetation (wild plants). IPM: IPM orchard, OP: organic palmette orchard, OG: organic goblet orchard. Vertical bars represent standard error of means.

The bee fauna visiting the flowers in the flowering mixture consisted of several taxa: *Apis mellifera*, wild bees of the genera *Andrena*, *Anthophora*, *Bombus*, *Eucera*, *Nomada*, *Hylaeus*, *Halictus*, *Lasioglossum*, *Sphecodes*, *Systropha*, *Xylocopa*, and the family Megachilidae. Honey bees were recorded visiting flowers of all broadleaved plant species of the sown mixture. Regarding the wild bee taxa composition in association to flower species, *V. faba* attracted *Eucera* spp. (mainly *E. nigrescens*), *A. plumipes*, *Bombus* spp. and *X. violaceae*; *Vicia sativa* attracted mainly *X. violaceae* and *Eucera* spp.; *L. sativus* attracted a few bees of the families Megachilidae and Halictidae; *E. sativa* attracted mainly Halictidae, *Anthophora* spp., and *Andrena* spp.; *C. sativum* attracted mainly *Hylaeus*, *Lasioglossum*, *Halictus*, *Andrena*. Bumblebees, *X. violaceae* and *Hylaeus* spp. were recorded only in the sown flowering patches and the mean numbers of *Anthophora* and *Eucera* visits on the sown mixture were higher compared to those of the wild plant patches (Figure 5).

Flowers of wild plant species also attracted several taxa of pollinators (plant sp.—pollinator taxa): *Veronica* spp.—*A. mellifera*, *Lasioglossum*, *Andrena*, Syrphidae; *Capsella bursa-pastoris*—*Lasioglossum*, *Andrena*, Bombyliidae, Syrphidae; *S. apetala*—*Lasioglossum* and Syrphidae; *C. draba*—*A. mellifera*, *Andrena*, *Lasioglossum*, Syrphidae; *Cerastium glomeratum* Thuill. (Caryophyllaceae)—*Lasioglossum*; *Ranunculus repens*—*A. mellifera* and *Lasioglossum*; *T. repens*—*A. mellifera*, *Eucera*, *Andrena*; *Taraxacum officinale* L. (Asteraceae)—Megachilidae (only one visit recorded); *Sonchus oleraceus* L. (Asteraceae)—*Lasioglossum* and Syrphidae; *C. irregularis*—mainly syrphid flies as well as few *Lasioglossum* and honey bees; *L. amplexicaule* (present only in the organic orchards)—honey bees and wild bees of the genera *Anthophora*, *Eucera* and *Andrena; Medicago arabica* L. (Fabaceae)—*Andrena*, *Eucera*, *Lasioglossum*; *C. arvensis*—*A. mellifera*, *S. curvicornis*, *Lasioglossum*, *Halictus*, *Andrena* (Figure 5). Most of these plant species were also present in the sown mixture patches (*Veronica* spp., *C. irregularis*, *C. bursa-pastoris*, *S. apetala*, *C. glomeratus*, *C. draba*), increasing their flower diversity.

**Figure 6:**
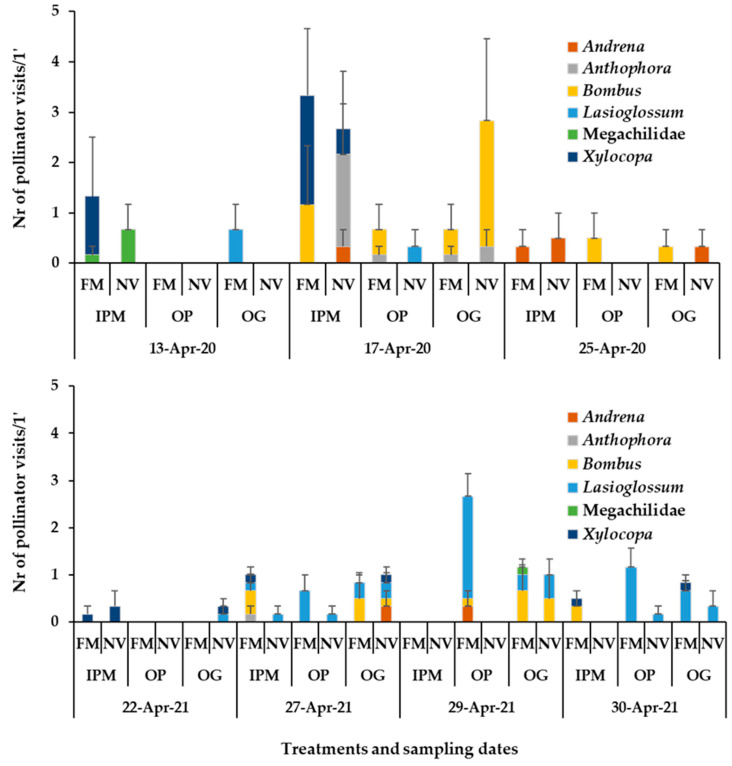
Mean numbers of wild bees recorded on apple flowers (1 min/6.4 m^2^) in 2020 and 2021. Wild bee taxa are indicated by different colour in each column. FM: sown flowering mixture; NV: natural vegetation (wild plants); IPM: IPM orchard, OP: organic palmette orchard, OG: organic goblet orchard. Vertical bars represent standard error of means.

**Figure 7:**
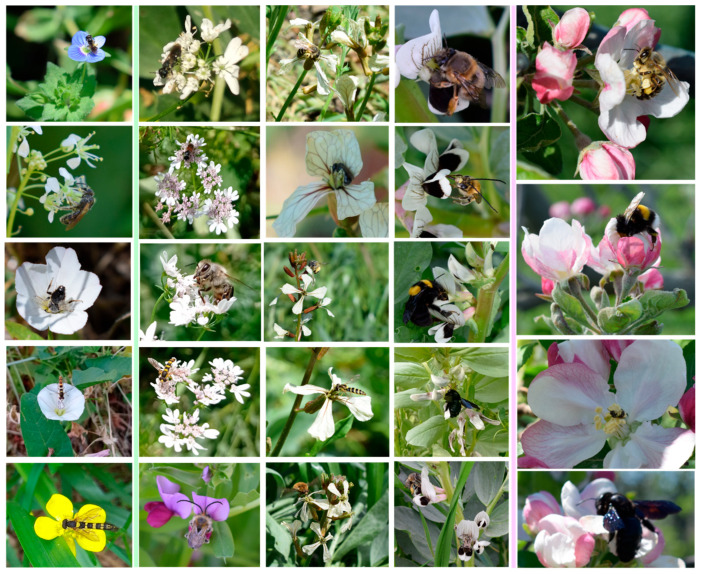
Insect pollinators in apple orchards in Tegea, Peloponnese, Greece, 2019–2021: **on wild plant flowers (1st column):** *Andrena* sp.—*Veronica persica*, *Lasioglossum* sp.—*C. draba*, *S. curvicornis*—*C. arvensis*, Syrphidae—*C. arvensis*, Syrphidae—*Ranunculus repens*; **on sown mixture flowers (2nd^—^4th column):** *Hylaeus* (Dentigera)—*C. sativum*, *Hylaeus variegatus*—*C. sativum*, *A. mellifera*—*C. sativum*, Syrphidae—*C. sativum*, *Eucera* sp.—*V. sativa*, *Andrena* sp.—*E. sativa*, *Lasioglossum* sp.—*E. sativa*, *Anthophora* sp.—*E. sativa*, Syrphidae—*E. sativa*, Bombyliidae—*E. sativa*, *A. plumipes*—*V. faba*, *E. nigrescens*—*V. faba*, *B. argilaceus*—*V. faba*, *X. violaceae*—*V. faba*, *A. plumipes* and *Eucera* sp.—*V. faba*; **on apple blossoms (5th column):** *A. mellifera-M. domestica*, *B. terrestris—M. domestica*, *Lasioglossum—M. domestica*, *X. violaceae—M. domestica.*

Wild bees recorded on apple flowers belong to the following taxa: *Andrena*, *Anthophora*, *Bombus* (e.g., *B. terrestris*, *B. argilaceus*), *Xylocopa*, *Lasioglossum* (e.g., *L. marginatum*), Megachilidae. The majority of wild bee visits in the sown patches of the OP orchard at the end of April in 2021 was from *Lasioglossum* spp. (Figure 6). Examples of insect pollinators recorded on the apple blossoms, the sown flowering mixture and the wild plants of the apple orchards are shown in Figure 7 and Table 2.

## 4. Discussion

Establishment of flower strips inside the crop or in field margins has been an agricultural practice in orchards for the support of functional biodiversity in agroecosystems, especially in relation to pollinators [106,107]. The present study tested the hypothesis that patches of flowering mixtures could enhance pollinators in apple orchards. In this respect, baseline recordings on the weed flora, before the intervention, were deemed necessary. The baseline fauna of pollinating insects on the wild plants of the orchards included few visits of *A. mellifera* mainly on *R. repens* and *C. arvensis* on IPM orchard, on *C. draba* and *C. arvensis* in OG orchard and on *C. arvensis* in OP orchard. The only wild bee visit on the wild plant patches in the baseline year was from *S. curvicornis* on flowers of *C. arvensis* in late June in the OG orchard. This observation aligns with the pollen preference of *S. curvicornis* as it is a specialist on plants of the Convolvulaceae family [16]. This species was also recorded in 2021 on *C. arvensis* on the wild plant patches, indicating the contribution of the weed flora in the conservation of wild pollinators. Syrphid flies were recorded on *Veronica* spp., *C. draba* and *R. repens* while the latter also attracted a few visits of bee flies (Bombyliidae).

The introduction of sown flowering patches in the two following years increased pollinator visitation on the groundcover of the apple orchards compared to the wild plants, while at the same time it attracted pollinators of diverse taxa. While similar findings were reported before [62,63], most of the published studies compare the pollinator visits on the flowers of the sown plants to regularly mown grassy field patches or margins [60,63], practically with null flowering resources. Thus, in the present study, the comparison is more challenging, yet the higher records of pollinator visits on the flower resources of the sown flowering patches vs. of the wild plant patches in the apple orchards were evident and statistically significant.

From the Fabaceae, the *V. faba* ‘Episkopi’ attracted *A. mellifera*, *Eucera* spp. (mainly *E. nigrescens*), *A. plumipes*, *Bombus* spp. (e.g., *B. argillaceus*, *B. terrestris*) and *X. violaceae*. Faba bean has been reported to attract honey bees and mainly long-tongued wild bees such as *Bombus*, *Xylocopa*, *A. plumipes*, as well as small-tongued bees because of its extrafloral nectar or through nectar robbing [55,108,109,110,111]. In addition, *V. sativa* attracted small numbers of *A. mellifera*, *X. violaceae*, *Eucera* spp. and fewer visits of bees in the genera *Andrena* and *Anthophora*. *Vicia sativa* started flowering approximately one month after *V. faba*, indicating that *V. sativa* extends the provision of legume flowering resources in the flowering mixture despite its low attraction of pollinators as it is also mentioned in [96]. *Lathyrus sativus* is cultivated as an annual spring crop due to the climatological conditions of Tegea plateau, which explains why it did not establish well when sown in the mixture as a winter crop. However, scattered plants of *L. sativus* PGI ‘Fava Feneou’ which reached flowering attracted a few bees of the families Megachilidae and Halictidae (genus *Lasioglossum*). *Lathyrus sativus* was the only plant species of the flowering mixture to attract bees of the family Megachilidae which has been reported to include pollinators of the apple crop [112,113,114,115]. Thus, its contribution to a spring flowering mixture should be well taken into consideration.

Despite the valuable source of foraging for pollinators that legumes can provide [92,93,95], their complex flower structures may not support the full range of pollinators present in agricultural landscapes as they might restrict accessibility of resources to pollinating taxa with short mouthparts [116,117,118,119]. Thus, it is necessary to include plants other than Fabaceae in the sown mixture to provide accessible floral resources for a diversity of pollinators. Brassicaceae and Apiaceae are two plant families that have been used in sown mixtures to attract insect pollinators [62].

From the Brassicaceae, *E. sativa* attracted wild bees of Halictidae (*Lasioglossum* spp., *Halictus* spp.) and honey bees. Lower visitation by *Anthophora* spp., *Eucera* spp., *Hylaeus* spp., *Andrena* (which has also been recorded on *E. sativa* flowers by [120]) were noted. *Eruca sativa* was the main plant of the flowering mixture to attract Syrphidae and Bombylidae. Syrphid flies are one of the most frequent visitors of *E. sativa* flowers [121] and have been reported to contribute to ecosystem services in agroecosystems through their supporting roles as crop pollinators and predators of pests [122].

From the Apiaceae, *A. graveolens* and *F. vulgare* had a poor establishment and did not reach flowering probably due to the prolonged low temperatures [123] after sowing (November–March), common in Tegea plateau, hence these species are probably not suitable for a winter sown mixture in areas with harsh weather conditions. *Coriandrum sativum* was the last species to bloom, extending the window of flowering period of the sown mixtures in the season when flower resources in the apple orchards were scarce (late May–June), hence adding extra value to the overall performance of the flowering mixtures. Coriander was attractive to both honey bees and wild bees. The wild bees which were recorded on *C. sativum* flowers in descending order belong to the genera *Hylaeus* (e.g., *H. variegatus*), *Lasioglossum*, *Halictus*, *Andrena*, *Sphecodes*, and have been reported before as pollinators of coriander [124,125]. Furthermore, *C. sativum* had many visits of syrphid flies in late May–June which have also been reported to visit this plant species by [57]. In the first year of introduction of sown flower patches (2020), coriander established only in the IPM orchard in patches where the interspecific competition with *V. faba* plants was lower. Considering that *V. faba* is a competitive species which can influence the ability of less competitive plants to persist and flower in a mixture [96], the seed percentage of *V. faba* in the sown mixture was almost halved to ensure the good establishment of coriander in all the experimental orchards in the second year of mixture estabishment (2021).

The number of honey bee and wild bee visits on the flowers of wild plant patches was low, yet the attracted pollinators included several genera. Previous studies have highlighted the role of weed flora in field margins in supporting pollinators and addressed the need to protect it and utilize this function in intensive farming systems [126,127]. However, the concerns of introducing species in the mixture which could spread into the crops and become noxious weeds still remain [128]. In fact, it has been suggested to include many plant families of the weed flora in the present study, such as Brassicaceae and Fabaceae, in wildflower seed mixes aiming to attract wild bees [129]. From Brassicaceae, *C. draba* in the present study attracted syrphids, honey bees and wild bees of the genera *Andrena* and *Lasioglossum*, while from Fabaceae, *T. repens* and *M. arabica* attracted honey bees and wild bees of *Eucera*, *Andrena* and *Lasioglossum*.

*Veronica hederifolia* and *V. persica* in the present study attracted *A. mellifera*, *Lasioglossum* (which have been also recorded on *V. persica* by [130]) and *Andrena*. Moreover, *V. hederifolia* has been reported to be an attractive nectar source for pollinators [127]. Here, *Veronica* spp. also attracted syrphid flies that have been documented on its flowers before [131]. *Stellaria apetala* attracted *Lasioglossum* sp. together with syrphid flies. Ara et al. [130] mention *Apis* spp. and *Lasioglosum* spp. on *Stellaria media* flowers, while wild bees of the genus *Lasioglossum* have also been reported on *Stellaria* spp. flowers by [132]. *Ranunculus repens* attracted small numbers of *A. mellifera* (which have been also recorded to visit *Ranunculus* sp. flowers by [130]) and *Lasioglossum*.

Similarly to this study, pollinator visits have been reported before on *C. bursa-pastoris* flowers by solitary bees and especialy *Andrena* spp. [133] and on *T. officinale* by megachilids [134,135]. *Lamium* sp. have been reported for visits by both long-tongued and short-tongued bees similarly to this case (*Anthophora*, *Eucera* and *Andrena*). However, *Bombus* spp. which are referred to as the main visiting pollinators of *Lamium* spp. [136] were not recorded here. *Convolulus arvensis* attracted mainly Halictidae [137] and honeybees. Convolvulaceae and in particular *C. arvensis* is an important floral resource for wild bees, especially those in the genus *Systropha*, [16,129]. However, *C. arvensis* and *C. bursa-pastoris* are listed among the most troublesome weeds in agriculture [138,139], which renders them undesirable in the field despite their capacity for attracting pollinators.

Overall, in the present study, the sown plants attracted pollinators from diverse genera with higher visitation of wild bees compared to the weed flora of the orchards. The superior effect of the sown patches was evident in all orchards and various conditions, i.e., rich (organic orchards) and poor (IPM) surrounding semi-natural habitats in respect to flower resources and nesting sites, different canopy architecture (goblette/palmete) and plant protection systems (organic/IPM). It is noteworthy that the usual practice in the area is avoiding the use of pesticides during the flowering period of the apple trees to avoid harming the pollinators visiting the apple flowers. Indeed, as Albrecht et al. [140] and McKerchar et al. [141] highlight, the sustainable use of pesticides in fields where conservation agronomic practices of wild pollinators are implemented is of outmost importance.

On the other hand, the contribution of the wild plant flower resources to attracting a diverse range of wild bee genera should not be ignored but exploited when planning groundcover management for the enhancement of pollinators. Wood et al. [142] conducted an experiment on agri-environment pollinator-friendly schemes and reported that the majority of bee species preferred wild plants that are not included in a flower-rich mixture, stressing the significance of the natural vegetation flowering resources inside an agroecosystem. In this study, most of the wild plant species (*Veronica* spp., *C. irregularis*, *C. bursa-pastoris*, *S. apetala*, *C. glomeratus*, *C. draba*) were also present in the sown mixtures, although in a lower density. Their presence increased the plant diversity of the sown flowering patches and subsequently the provided floral resources. The differences in the pollinator abundance and the genera recorded among the apple orchards suggest that groundcover management recommendations must be site-specific to ensure the long-term availability of diverse floral resources for pollinators [134].

To return to our hypothesis, that sown flowering patches could enhance pollinators’ presence and diveristy also to the benefit of the crop, *A. mellifera* was the most frequent pollinator on the apple blossoms in all years. The lower range of means of honey bee visits during apple full bloom in the baseline year compared to the two following years is attributed to the prolonged rainy and cloudy weather during the short flowering period (10 days) of ‘Pilafa’ in Tegea plateau in 2019 and is not linked with the groundcover management. However, when the sown flower patches were present in the orchards, visits of wild bees were also recorded on apple blossoms. Previous studies have stated that honey bees outnumber wild bees in apple orchards [143,144] or that wild pollinators were not observed to visit apple flowers and honey bee was the only apple flower visitor [145]. Nevertheless, published studies highlight the importance of management for diverse pollinator communities to apple crop pollination, even in the presence of large populations of managed honey bees, which may also decrease reliance on managed honey bees for pollination services and enhance crop yields [146,147]. The common wild bee taxa recorded on apple flowers and on the sown/wild plant patches include Halictidae, Megachilidae, *Andrena*, *Anthophora* as well as *Bombus* and *Xylocopa* (only in sown mixture). In 2020, *Bombus* and *Xylocopa* visits on apple flowers could be associated with their attractance by *V. faba* in the sown patches. In 2021, higher visitation of *Lasioglossum* spp. on the apple flowers of the trees adjacent to the sown mixture could be attributed to *E. sativa* plants which attracted high numbers of *Lasioglossum* spp. However, there is no clear pattern to link wild bee visitation on apple flowers and the sown patches. Lowe et al. [148] and Albrecht et al. [140] conducted review meta-analyses which showed that flower plantings in agroecosystems are highly effective at increasing pollinator richness and abundance in the intervention area, but the influence of these plantings on crop pollination and yield is inconsistent.

The landscape elements around the apple orchards could have also influenced the pollinator taxa that visited the apple flowers [149]. For example, the organic goblet orchard attracted *Bombus* spp. in trees adjacent to both sown mixture and wild plant patches. The flowering resources on undisturbed land around the organic goblet orchard included *T. repens*, *M. polymorpha*, *V. villosa*, all natural vegetation of Fabaceae plant species, which are the major pollen source for most bumblebee species [45], as well as wild forest trees and bushes which prevent human passage and might contribute to the formation of protected pollinator nesting sites. This might explain the presence of *Bombus* spp. on apple flowers regardless of the ground management inside the organic goblet orchard in 2020 and 2021. Földesi et al. [145] mention that the maintenance of semi-natural habitats within 500 m around apple orchards is highly recommended to enhance wild pollinator communities and apple production. Moreover, Gervais et al. [150] also report that landscape enhancements improve bumble bee queen presence and diversity in apple orchards and should therefore be considered by growers as a means to enhance and ensure the pollination and diversity of beneficial insects in their orchards. Therefore, policy measures for pollinators should reffer not only to agroecosystems but to various landscapes and neighbouring specialised habitats as maintaining species diversity is crucial in providing ecosystem resilience. Furthermore, the environental policies on pollinators should have a holistic ecosystem approach taking into acount the fact that ecosystem service management, such as establishment of sown flowering patches in perenial crops, does not equal biodiversity conservation, but that these terms are interlinked [151].

## 5. Conclusions

Honey bee (*A. mellifera*) is the main pollinator of the apple crop in ‘Pilafa’ apple orchards in Tegea, Peloponnese, Greece. Wild plants in the groundcover provided floral supplies for different genera of pollinators, but these were not notably attractive to pollinators as indicated by the small number of visits mainly of honey bees, Halictidae and Syrphidae. The introduction of sown flowering patches of *V. faba*, *V. sativa*, *E. sativa*, *C. sativum* in mixtures attracted honey bees and wild bees in greater numbers and more diverse wild bee taxa indicating the importance of the floral abundance and diversity as well as flowering period of the resources for the pollinating insects. The plant species *E. sativa* and *V. faba* of the sown mixture attracted high numbers and diverse pollinator fauna (*A. mellifera*, *Eucera* spp., *Anthophora* spp., *Bombus* spp., *X. violaceae*, Halictidae, *Andrena* spp.) which also visited apple flowers except for *Eucera* spp., whereas *C. sativum* provided resources for pollinators (mainly *Hylaeus* spp., Halictidae, *Andrena* spp. and honey bees) in a period when the available flowering reservoirs in the apple orchards were scarce. The results are valuable for a better understanding of the flora–pollinator fauna relationships in apple orchards and for the design of future sustainable management strategies mainly in relation to ecosystem pollination services in this crop. The tested sown mixture, including the legume landraces, in patches provides floral sources for honey bees, wild bees, syrphid flies and bee flies in the studied area and can serve as a good agricultural tool/practice to attract insect pollinators and potentially enhance pollination if adjusted appropriately to meet site-specific parameters in respect to pollinator conservation and exploitation of wild flora in agroecosystems.

## Figures and Tables

**Table 1:** Selected plant species used in sown seed mixtures in patches at the groundcover of apple orchards and seed percentage of each species in the two experimental years.

Family	Plant Species	Seed Percentage (%)	
		Year	
		2020	2021
Apiaceae	*Anethum graveolens* L.	15	10
Apiaceae	*Coriandrum sativum* L.	15	20
Apiaceae	*Foeniculum vulgare* Mill.	-	10
Brassicaceae	*Eruca sativa* Mill.	15	25
Fabaceae	*Lathyrus sativus* L. (PGI ‘Fava Feneou’)	15	-
Fabaceae	*Vicia faba* L. (landrace ‘Episkopis’)	15	7
Fabaceae	*Vicia sativa* L.	15	23
Poaceae	*Triticum aestivum* L. (landrace ‘Zoolitsa’)	15	-
Poaceae	*Triticum aestivum* L. (landrace ‘Asprositi’)	-	5

**Table 2:** Hymenopterous pollinators and associated flowering in sown flowering mixtures and the natural vegetation (wild plants) in apple orchards in Tegea, Peloponnese, Greece, 2019–2021.

Family	Genus	Species	Associated Plants
Andrenidae	*Andrena*	*Andrena* sp1 (♀) *Andrena* sp2 (♀) *Andrena* sp3 (♀) *Andrena* sp4 (♀) *Andrena* sp5 (♀) *Andrena* sp6 (♀,♂) *Andrena* sp7 (♀) *Andrena* sp8 (♀) *Andrena* sp9 (♀) *Andrena* sp10 (♀) *Andrena* sp11 (♂) *Andrena* sp12 (♀,♂)	*Taraxacum* sp., *Daucus carota* *Gallium aparine* *Veronica* sp. *Stellaria apetala* *Malus domestica* FM, *Medicago arabica* *Eruca. sativa* *Trifolium repens* *Coriandrum sativum* *Coriandrum sativum* *Veronica* sp. FM, *Coriandrum sativum*, *Cardaria draba*
Apidae	*Apis*	*A. mellifera* (♀)	*M. domestica*, *Vicia faba*, *Coriandrum sativum*, *Veronica* spp., *E. sativa*, *Ranunculus repens*, *Calepina irregularis*, *Lamium amplexicaule*, *Cardaria draba*, *Triffolium repens*, *Convolvulus arvense*, *Raphanus* sp.
	*Anthophora*	*A. plumipes* (♀) *Anthophora* spp. (♀)	*Vicia faba*, *Eruca sativa* *Eruca sativa*, *Lamium amplexicaule*
	*Bombus*	*B. argillaceus* (♀) *B. terrestris* (♀,♂) *Bombus* sp1 (♀) *Bombus* sp2 (♀)	*Vicia faba* *Vicia faba*, *Malus domestica* *Malus domestica* *Vicia faba*
	*Eucera*	*E. nigrescens* (♀,♂) *Eucera* sp1 (♂) *Eucera* sp2 (♂)	FM, *Torilis* sp., *V. villosa*, *V. faba*, *E. sativa* *Medicago arabica* *Eruca sativa*
	*Nomada*	*Nomada* sp. (♀)	NV
	*Xylocopa*	*X. violaceae* (♀,♂)	*Vicia faba*, *Vicia sativa*
Colletidae	*Hylaeus*	*H. variegatus* (♀) *Hylaeus* sp1 (♀,♂) *Hylaeus* sp2 (♀)	*Coriandrum sativum* *Coriandrum sativum* *Eruca sativa*
Halictidae	*Halictus*	*Halictus* sp1 (♀) *Halictus* sp2 (♀) *Halictus* sp3 (♀)	*Crepis* sp. *Coriandrum sativum* *Coriandrum sativum*
	*Lasioglossum*	*L. marginatum* (♀) *Lasioglossum* sp1 (♀) *Lasioglossum* sp2 (♀) *Lasioglossum* sp3 (♀) *Lasioglossum* sp4 (♀) *Lasioglossum* sp5 (♀) *Lasioglossum* sp6 (♀) *Lasioglossum* sp7 (♀) *Lasioglossum* sp8 (♀) *Lasioglossum* sp9 (♀) *Lasioglossum* sp10 (♀) *Lasioglossum* sp11 (♀) *Lasioglossum* sp12 (♀) *Lasioglossum* sp13 (♀)	*Malus domestica* *Veronica* sp., *Sonchus oleraceus* *Eruca sativa*, *Veronica* sp. *Lathyrus sativus* *Eruca sativa* *Malus domestica* *Veronica* sp. *Eruca sativa* *Capsella bursa-pastoris* *Medicago arabica* *Ranunculus* repens *Coriandrum sativum* *Convolvulus arvense* NV
	*Systropha*	*S. curvicornis* (♀,♂)	*Convolvulus arvense*
	*Sphecodes*	*Sphecodes* sp. (♀)	*Coriandrum sativum*

Column ‘Associated plants’: FM = sown flowering mixture; NV = natural vegetation (wild plants).

## Data Availability

Not applicable.

## Supplementary Materials

The following supporting information can be downloaded at: https://www.mdpi.com/article/10.3390/insects14020208/s1, Figure S1: Experimentation sites of two organic orchards (OP: organic palmette orchard, OG: organic goblet orchard) and one IPM orchard (37.43705, 22.46942; 37.43658, 22.46895; 37.4415, 22.40492) in Tegea plateau, Peloponnese, Greece, and example of the layout of the sown flowering mixture patches (FM) (pink blocks) and the natural vegetation patches (NV) (green blocks) in the apple orchards where the presence of pollinators was recorded; Figure S2: Meteorological data [Temperature (°C), rain (mm), wind speed (km/hr)] for years 2019, 2020, 2021 from the National Observatory of Athens. Meteorological station ‘Tripoli (LG83)’, elevation: 646 m latitude: 37°30′34″ N, longitude: 22°25′04″ E; Table S1: Mean number of honey bee and wild bee visits/patch/1′ (±s.e.m.) in the sown flowering mixture (FM) patches and the natural vegetation (NV) patches of groundcover, at three apple orchards (IPM, OP, OG), in three consecutive years 2019, 2020, 2021 (recordings from April to June); Table S2: Mean number of honey bee and wild bee visits/plot/1′ (±s.e.m.) on the apple tree blossoms adjacent to the sown flowering mixture (FM) patches and the natural vegetation (NV) patches of groundcover, at three apple orchards (IPM, OP, OG), in years 2019, 2020, 2021 (recordings from April to June).
