# Supplementary material for: Do Patches of Flowering Plants Enhance Insect Pollinators in Apple Orchards?
Source: Insects. 2023 Feb 19;14(2):208. doi: 10.3390/insects14020208 (PMC9960344; doi:10.3390/insects14020208)
Supplement: Supplementary file 1 [file insects-14-00208-s001.zip › insects-2213012-supplementary.pdf]

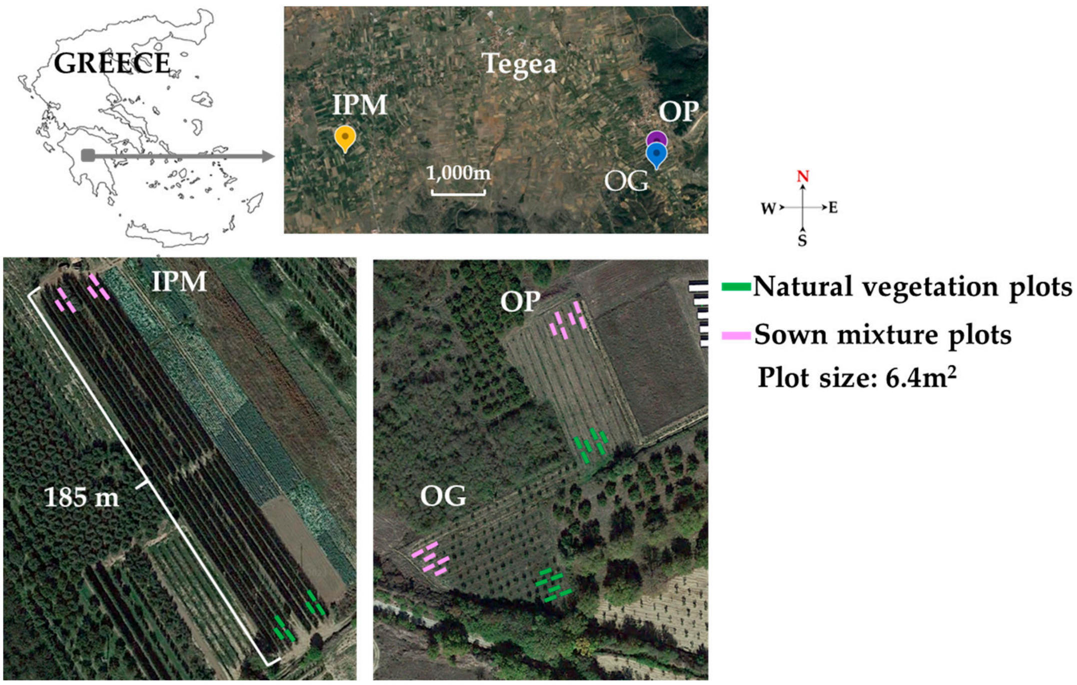

Figure S1: Experimentation sites of two organic orchards (OP: Organic palmette orchard, OG: Organic goblet orchard) and one IPM orchard (37.43705, 22.46942; 37.43658, 22.46895; 37.4415, 22.40492) in Tegea plateau, Peloponnese, Greece, and example of the layout of the sown flowering mixture patches (FM) (pink blocks) and the natural vegetation patches (NV) (green blocks) in the apple orchards where the presence of pollinators was recorded.

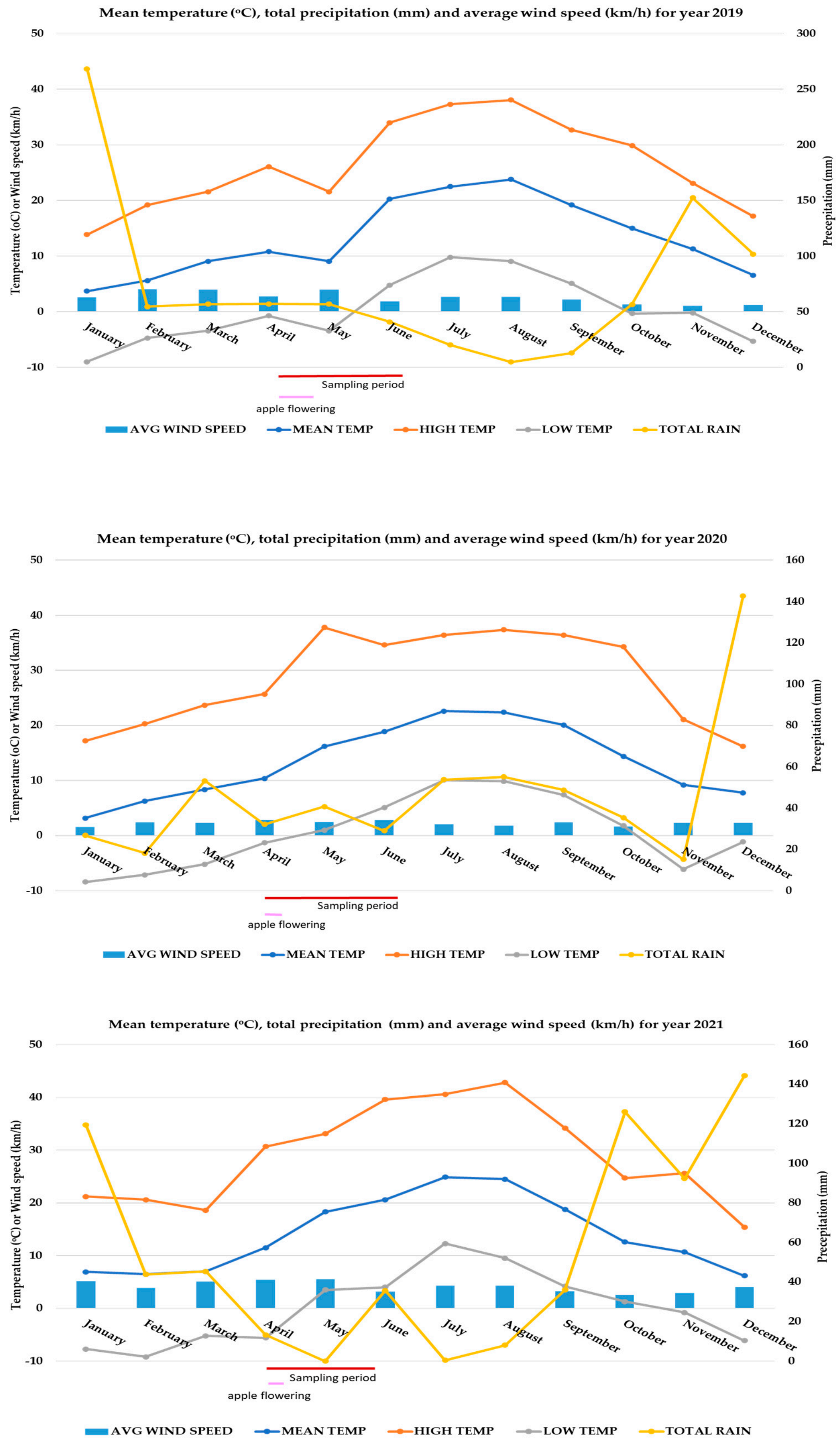

Figure S2: Meteorological data [Temperature (°C), rain (mm), wind speed (km/hr)] for years 2019, 2020, 2021 from the National Observatory of Athens. Meteorological station "Tripoli (LG83)", elevation: 646m latitude: 37° 30' 34'' N, longitude: 22° 25' 04'' E.

Table S1. Mean number of honey bee and wild bee visits/patch/1′ (± s.e.m.) in the sown flowering mixture (FM) patches and the natural vegetation (NV) patches of groundcover, at three apple orchards (IPM, OP, OG), in three consecutive years 2019, 2020, 2021 (recordings from April to June).

**Honey bees**						
	**Baseline Year 2019**					
Orchard /Date	IPM (2 sites)		OP		OG	
	Site a	Site b				
01 May 2019	0.00 ± 0.00	0.17 ± 0.17	0.00 ± 0.00		0.00 ± 0.00	
17 May 2019	0.00 ± 0.00	0.00 ± 0.00	0.00 ± 0.00		0.33 ± 0.33	
22 June 2019	0.17 ± 0.17	0.00 ± 0.00	0.33 ± 0.21		0.33 ± 0.21	
2-way ANOVA	Orchard: $F_{2,63}$=1.34, p=0.2691; Date: $F_{2,63}$=2.19, p= 0.1204; Orchard *Date: $F_{4,63}$=1.17, p= 0.3313					
	**Year 2020**					
Orchard /Treatment/ Date	IPM		OP		OG	
	FM	NV	FM	NV	FM	NV
13 Apr. 2020	6.50 ± 1.23 Aa	0.00 ± 0.00 Bb	5.17 ± 0.79 Aa	0.00 ± 0.00 Ba	3.50 ± 0.43 Aa	0.00 ± 0.00Bab
17 Apr. 2020	4.50 ± 1.43 Aa	0.00 ± 0.00 Bb	1.50 ± 0.96 Ab	0.00 ± 0.00 Aa	2.83 ± 0.91 Aab	0.33 ± 0.33Bab
25 Apr. 2020	0.00 ± 0.00 Ab	0.00 ± 0.00 Ab	0.00 ± 0.00 Ab	0.67 ± 0.42 Aa	1.00 ± 0.45 Aab	0.00 ± 0.00Bab
08 May 2020	1.00 ± 0.26 Ab	3.00 ± 1.13 Aa	1.17 ± 0.54 Ab	0.00 ± 0.00 Aa	2.83 ± 1.49 Aab	0.00 ± 0.00Aab
16 May 2020	1.17 ± 0.65 Ab	0.00 ± 0.00 Ab	0.00 ± 0.00 Ab	0.00 ± 0.00 Aa	0.50 ± 0.34 Aab	0.17 ± 0.17Aab
25 May 2020	0.00 ± 0.00 Ab	0.00 ± 0.00 Ab	0.00 ± 0.00 Ab	0.00 ± 0.00 Aa	0.00 ± 0.00 Ab	1.00 ± 0.63 Aa
12 June 2020	0.00 ± 0.00 Ab	0.00 ± 0.00 Ab	0.00 ± 0.00 Ab	0.00 ± 0.00 Aa	0.00 ± 0.00 Ab	0.00 ± 0.00 Ab
26 June 2020	0.00 ± 0.00 Ab	0.00 ± 0.00 Ab	0.00 ± 0.00 Ab	0.00 ± 0.00 Aa	0.00 ± 0.00 Ab	0.00 ± 0.00 Aab
2-way ANOVA	**Groundcover: F1,80=19.40, p<0.0001; Date: F7,80 =10.12, p <0.0001; Groundcover*Date: F7,80 =11.78, p<0.0001**		**Groundcover: $F_{1,70}$= 25.46, p<0.0001; Date: $F_{6,70}$=11.81, p <0.0001; Groundcover*Date: $F_{6,70}$=13.54, p<0.0001**		**Groundcover: $F_{1,70}$=20.52, p<0.0001; Date: $F_{6,70}$=3.30, p <0.0001; Groundcover*Date: $F_{6,70}$=4.74, p<0.0001**	
	**Year 2021**					
Orchard /Treatment/ Date	IPM		OP		OG	
	FM	NV	FM	NV	FM	NV
09 Apr. 2021	0.00 ± 0.00 Ab	0.00 ± 0.00 Aa	0.83 ±0.48 Ad	0.17 ± 0.17 Aa	1.00 ± 0.37 Aab	0.33 ± 0.33 Aa
15 Apr. 2021	0.50 ± 0.34 Aab	0.00 ± 0.00 Aa	1.33 ± 0.33 Ad	0.50 ± 0.50 Aa	1.50 ± 0.62 Aab	0.00 ± 0.00 Ba
22 Apr. 2021	0.17 ± 0.17 Aab	0.17 ± 0.17 Aa	0.67 ± 0.33 Ad	0.00 ± 0.00 Aa	1.33 ± 0.49 Aab	0.00 ± 0.00 Ba
27 Apr. 2021	0.17 ± 0.17 Aab	0.00 ± 0.00 Aa	1.50 ± 0.22 Acd	0.00 ± 0.00 Ba	1.00 ± 0.52 Aab	0.00 ± 0.00 Aa
29 Apr. 2021	0.00 ± 0.00 Ab	0.00 ± 0.00 Aa	2.17 ± 0.60 Abcd	0.17 ± 0.17 Ba	0.83 ± 0.31 Ab	0.00 ± 0.00 Ba
30 Apr. 2021	0.00 ± 0.00 Ab	0.00 ± 0.00 Aa	2.67 ± 0.56 Aabcd	0.17 ± 0.17 Ba	2.33 ± 0.71 Aab	0.00 ± 0.00 Ba
6 May 2021	2.00 ± 1.03 Aa	0.33 ± 0.21 Aa	5.33 ± 1.41 Aa	0.33 ± 0.33 Ba	2.33 ± 0.71 Aab	0.33 ± 0.33 Ba
14 May 2021	0.17 ± 0.17 Aab	0.00 ± 0.00 Aa	4.33 ± 0.80 Aabc	0.00 ±0.00 Ba	3.67 ± 0.92 Aa	1.00 ± 0.68 Ba

20 May 2021	1.00 ± 0.63 Aab	0.00 ± 0.00 Aa	4.83 ± 0.60 Aab	0.33 ± 0.21 Ba	2.83 ± 0.65 Aab	0.00 ± 0.00 Ba
28 May 2021	0.00 ± 0.00 Ab	0.00 ± 0.00 Aa	0.50 ± 0.34 Ad	0.67 ± 0.33 Aa	0.50 ± 0.50 Ab	0.50 ± 0.50 Aa
8 June 2021	0.00 ± 0.00 Ab	0.00 ± 0.00 Aa	0.00 ± 0.00 Ad	0.50 ± 0.34 Aa	0.17 ± 0.17 Ab	0.17 ± 0.17 Aa
2-way ANOVA	Groundcover: $F_{1,110}$=7.04, p=0.0091; Date: $F_{10,110}$=3.17, p=0.0013; Groundcover*Date: $F_{10,110}$=1.87, p=0.055		**Groundcover: $F_{1,110}$=91.22, p< 0.0001; Date: $F_{10,110}$=7.37, p<0.0001; Groundcover*Date: $F_{10,110}$=8.16, p<0.0001**		**Groundcover: $F_{1,110}$=49.35, p<0.0001; Date: $F_{10,110}$=3.59, p= 0.0004; Groundcover*Date: $F_{10,110}$=2.32, p=0.0161**	
**Wild bees**						
	**Year 2019**					
Orchard /Date	IPM (2 sites)		OP		OG	
	Site a	Site b				
01 May 2019	0.00 ± 0.00	0.00 ± 0.00	0.00 ± 0.00		0.00 ± 0.00	
17 May 2019	0.00 ± 0.00	0.00 ± 0.00	0.00 ± 0.00		0.00 ± 0.00	
22 June 2019	0.00 ± 0.00	0.00 ± 0.00	0.00 ± 0.00		0.17 ± 0.17	
2-way ANOVA	Orchard: $F_{2,63}$=1.57, p=0.215; Date: $F_{2,63}$=1.68, p=0.1946; Orchard *Date: $F_{4,63}$=1.57, p=0.1919					
	**Year 2020**					
Orchard /Treatment/ Date	IPM		OP		OG	
	FM	NV	FM	NV	FM	NV
13 Apr. 2020	4.00 ± 1.15 Aab	0.00 ± 0.00 Ba	9.00 ± 1.13 Aa	0.33 ± 0.21 Bb	6.00 ± 0.73 Abc	0.5 ± 0.34 Ba
17 Apr. 2020	4.83 ± 0.79 Aab	0.00 ± 0.00 Ba	11.17± 2.89 Aa	0.88 ± 0.48 Bb	2.17 ± 3.13 Aa	0.00 ± 0.00 Ba
25 Apr. 2020	7.33 ± 2.12 Aa	0.00 ± 0.00 Ba	5.17 ± 1.19 Aab	2.50 ± 0.56 Aa	8.67 ± 1.52 Aab	0.50 ± 0.50 Ba
08 May 2020	2.83 ± 0.60 Aab	0.83 ± 0.54 Ba	5.17 ± 2.66 Aab	0.50 ± 0.34 Ab	2.00 ± 0.52 Acd	0.17 ± 0.17 Ba
16 May 2020	2.50 ± 0.72 Aab	0.67 ± 0.49 Aa	1.17 ± 0.98 Ab	0.17 ± 0.17 Ab	0.33 ± 0.33 Acd	0.17 ± 0.17 Aa
25 May 2020	1.83 ± 1.01 Ab	0.00 ± 0.00 Aa	1.00 ± 0.82 Ab	0.17 ± 0.17 Ab	0.83 ± 0.40 Acd	1.17 ± 0.98 Aa
12 June 2020	1.83 ± 0.54 Aab	0.00 ± 0.00 Ba	0.00 ± 0.00 Ab	0.00 ± 0.00 Ab	0.00 ± 0.00 Ad	0.00 ± 0.00 Aa
26 June 2020	3.00 ± 1.21 Aab	0.00 ± 0.00 Ba	0.00 ± 0.00 Ab	0.00 ± 0.00 Ab	0.00 ± 0.00 Ad	0.00 ± 0.00 Aa
2-way ANOVA	**Groundcover: $F_{1,80}$=66.59, p<0.0001; Date: $F_{7,80}$=2.38, p= 0.0285; Groundcover*Date: $F_{7,80}$=2.93, p=0.0089**		**Groundcover: $F_{1,70}$=38.62, p< 0.0001; Date: $F_{6,70}$=7.25, p<0.0001; Groundcover*Date: $F_{6,70}$=5.63, p<0.0001**		**Groundcover: $F_{1,70}$=51.82, p< 0.0001; Date: $F_{6,70}$=10.58, p<0.0001; Groundcover*Date: $F_{6,70}$=11.23, p<0.0001**	
	**Year 2021**					
Orchard /Treatment/ Date	IPM		OP		OG	
	FM	NV	FM	NV	FM	NV
09 Apr. 2021	3.67 ± 1.43 Aab	0.00 ± 0.00 Bb	6.67 ± 2.36 Aabc	0.67 ± 0.33 Bab	4.50 ± 1.18 Aa	0.67 ± 0.33 Ba
15 Apr. 2021	5.67 ± 1.12 Aab	0.00 ± 0.00 Bb	3.17 ± 0.60 Abcd	0.67 ± 0.33 Bab	3.50 ± 0.92 Aabc	0.17 ± 0.17 Ba
22 Apr. 2021	4.00 ± 0.68 Aab	0.00 ± 0.00 Bb	3.50 ± 0.85 Aabcd	1.17 ± 0.31 Bab	3.50 ± 0.50 Aabc	0.33 ± 0.21 Ba
27 Apr. 2021	3.33 ± 0.67 Aab	0.17 ± 0.17 Bb	3.00 ± 0.63 Abcd	0.17 ± 0.17 Bb	2.00 ± 0.82 Aabcde	0.50 ± 0.50 Aa
29 Apr. 2021	3.33 ± 0.21 Aab	0.17 ± 0.17 Bb	2.33 ± 0.56 Acd	1.83 ± 0.31 Aa	1.33 ± 0.49 Abcde	1.00 ± 0.37 Aa

30 Apr. 2021	2.50 ± 1.02 Aab	1.33 ± 0.61 Aa	1.17 ± 0.40 Ad	0.17 ± 0.17 Bb	1.17 ± 0.60 Abcde	0.17 ± 0.17 Aa
06 May 2021	1.67 ± 0.76 Ab	0.00 ± 0.00 Bb	2.33 ± 0.61 Acd	0.83 ± 0.40 Aab	0.33 ± 0.33 Ade	0.17 ± 0.17 Aa
14 May 2021	2.17 ± 0.65 Aab	0.17 ± 0.17 Bb	0.83 ± 0.40 Ad	0.33 ± 0.21 Aab	0.50 ± 0.34 Acde	0.00 ± 0.00 Aa
20 May 2021	1.67 ± 0.61 Ab	0.00 ± 0.00 Bb	3.00 ± 0.68 Abcd	0.00 ± 0.00 Bb	0.00 ± 0.00 Ae	0.00 ± 0.00 Aa
28 May 2021	3.83 ± 1.01 Aab	0.67 ± 0.33 Bab	8.17 ± 1.28 Aa	0.17 ± 0.17 Bb	4.17 ± 0.65 Aab	0.33 ± 0.33 Ba
08 June 2021	6.50 ± 1.48 Aa	0.00 ± 0.00 Bb	7.67 ± 0.76 Aab	1.17 ± 0.65 Bab	3.33 ± 0.61 Aabcd	0.00 ± 0.00 Ba
2-way ANOVA	**Groundcover: $F_{1,110}$=122.74, p<0.0001; Date: $F_{10,110}$=2.38, p=0.0135; Groundcover*Date: $F_{10,110}$=2.95, p=0.0025**		**Groundcover: $F_{1,110}$=101.27, p<0.0001; Date: $F_{10,110}$=6.39, p<0.0001; Groundcover*Date: $F_{10,110}$=6.06, p<0.0001**		**Groundcover: $F_{1,110}$=79.85, p<0.0001; Date: $F_{10,110}$=6.09, p<0.0001; Groundcover*Date: $F_{10,110}$=5.01, p<0.0001**	

Capital letters indicate significant differences between treatments while small letters indicate significant differences between assessment dates. Bold fonts indicate significant statistical differences in 2 way-ANOVA.

IPM: IPM orchard, OP: organic palmette orchard, OG: organic goblet orchard.

Table S2. Mean number of honey bee and wild bee visits/plot/1' (± s.e.m.) on the apple tree blossoms adjacent to the sown flowering mixture (FM) patches and the natural vegetation (NV) patches of groundcover, at three apple orchards (IPM, OP, OG), in years 2019, 2020, 2021 (recordings from April to June).

**Honey bees**						
	**Baseline Year 2019**					
Orchard /Date	IPM (2 sites)		OP		OG	
	Site a	Site b				
23 Apr. 2019	11.50 ± 1.84	11.00 ± 1.50	9.17 ± 4.80		10.00 ± 1.15	
01 May 2019	2.50 ±1.08	3.33 ± 0.84	4.83 ± 0.94		10.33 ± 1.68	
2-way ANOVA	Orchard: $F_{3,40}$=1.06, p=0.3763; **Date: $F_{1,40}$=11.67, p=0.0015**; Orchard *Date: $F_{3,40}$=1.89, p=0.1465					
	**Year 2020**					
Orchard /Treatment/ Date	IPM		OP		OG	
	FM	NV	FM	NV	FM	NV
13 Apr. 2020	4.33 ± 1.45 Ab	6.33 ± 1.02 Ab	1.83 ± 0.79 Ac	2.33 ± 0.55 Ac	3.17 ± 1.42 Ab	1.33 ± 0.71 Ab
17 Apr. 2020	36.17 ± 2.71 Aa	37.00 ± 5.85 Aa	9.83 ± 0.98 Bb	18.83 ± 2.08 Ab	15.33 ± 1.83 Aa	17.16 ± 2.38 Aa
25 Apr. 2020	11.50 ± 1.87 Ab	8.00 ± 0.85 Ab	31.00 ± 3.50 Aa	27.66 ± 2.01 Aa	26.33 ± 5.58 Aa	23.66 ± 6.05 Aa
2-way ANOVA	**Groundcover: $F_{1,30}$=0.009, p=0.9248; Date: $F_{2,30}$=69.99, p<0.0001**; Groundcover*Date: $F_{2,30}$=0.51, p=0.6034		Groundcover: $F_{1,30}$=1.68, p= 0.2042; **Date: $F_{2,30}$=99.02, p<0.0001; Groundcover*Date: $F_{2,30}$=5.29, p=0.0107**		Groundcover: $F_{1,30}$=0.09, p= 0.7669; **Date: $F_{2,30}$=19.87, p<0.0001**; Groundcover*Date: $F_{2,30}$=0.21, p=0.8068	
	**Year 2021**					
v/Treatment/ Date	IPM		OP		OG	
	FM	NV	FM	NV	FM	NV
15 Apr. 2021	0.83 ± 0.54 Ac	0.5 ± 0.5 Ac	0.17 ± 0.17 Ac	0.33 ± 0.33 Ab	0.00 ± 0.00 b	0.00 ± 0.00 b

22 Apr. 2021	20.50 ± 3.05 Aa	25.83 ± 2.37 Aa	0.33 ± 0.33 Ac	0.66 ± 0.42 Ab	10.16 ± 1.85 b	5.50 ± 1.23 b
27 Apr. 2021	21.33 ± 1.72 Aa	22.83 ± 1.81 Aa	11.00 ± 2.50 Ab	14.66 ± 2.53 Aa	38.00 ± 4.75 a	34.16 ± 5.27 a
29 Apr. 2021	18.5 ± 0.42 Aab	15.5 ± 1.47 Ab	22.83 ± 3.39 Aa	12.67 ± 2.49 Ba	36.17 ± 3.55 a	30.67 ± 2.80 a
30 Apr. 2021	12.0 ± 1.06 Ab	5.33 ± 0.91 Bc	15.33 ± 2.67 Aab	10.66 ± 1.80 Aa	34.50 ± 6.85 a	29.67 ± 2.95 a
2-way ANOVA	Groundcover: $F_{1,50}$=0.38, p=0.5382; **Date: $F_{4,50}$=69.73, p<0.0001; Groundcover*Date: $F_{4,50}$=3.93, p=0.0074**		Groundcover: $F_{1,50}$=2.75, p=0.1029; **Date: $F_{4,50}$=31.04, p<0.0001; Groundcover*Date: $F_{4,50}$=3.51, p=0.0132**		Groundcover: $F_{1,50}$=2.69, p=0.1068; **Date: $F_{4,50}$=42.38, p<0.0001**; Groundcover*Date: $F_{4,50}$=0.18, p=0.9467	
**Wild bees**						
	**Year 2019**					
Orchard /Date	IPM (2 sites)		OP		OG	
	Site a	Site b				
23 Apr. 2019	0.00 ± 0.00	0.00 ± 0.00	0.00 ± 0.00		0.00 ± 0.00	
01 May 2019	0.00 ± 0.00	0.00 ± 0.00	0.00 ± 0.00		0.00 ± 0.00	
2-way ANOVA	n.a.					
	**Year 2020**					
Orchard /Treatment/ Date	IPM		OP		OG	
	FM	NV	FM	NV	FM	NV
13 Apr. 2020	1.33 ± 1.14 a	0.66 ± 0.49 a	0.00 ± 0.00	0.00 ± 0.00	0.66 ± 0.49	0.00 ± 0.00
17 Apr. 2020	3.33 ± 1.45 a	2.66 ± 1.54 a	0.67 ± 0.49	0.33 ± 0.33	0.67 ± 0.67	2.83 ± 1.55
25 Apr. 2020	0.33 ± 0.33 a	0.50 ± 0.50 a	0.50 ± 0.50	0.00 ± 0.00	0.33 ± 0.33	0.33 ± 0.33
2-way ANOVA	Groundcover: $F_{1,30}$=0.21, p= 0.6481; **Date: $F_{2,30}$=3.43, p=0.0452**; Groundcover*Date: $F_{2,30}$=0.10, p=0.8976		Groundcover: $F_{1,30}$=1.14, p=0.2928; Date: $F_{2,30}$=1.23, p=0.3042; Groundcover*Date: $F_{2,30}$=0.32, p=0.7278		Groundcover: $F_{1,30}$=0.67, p=0.4182; Date: $F_{2,30}$=2.40, p=0.1075; Groundcover*Date: $F_{2,30}$=1.97, p=0.1568	
	**Year 2021**					
Orchard /Treatment/ Date	IPM		OP		OG	
	FM	NV	FM	NV	FM	NV
22 Apr. 2021	0.17 ± 0.17	0.33 ± 0.33	0.00 ± 0.00 Ab	0.00 ± 0.00 Aa	0.00 ± 0.00	0.33 ± 0.33
27 Apr. 2021	1.00 ± 0.44	0.17 ± 0.17	0.67 ± 0.33 Ab	0.17 ± 0.17 Aa	0.83 ± 0.65	1.00 ± 0.26
29 Apr. 2021	0.00 ± 0.00	0.00 ± 0.00	2.67 ± 0.71 Aa	0.00 ± 0.00 Ba	1.17 ± 0.83	1.00 ± 0.68
30 Apr. 2021	0.50 ± 0.34	0.00 ± 0.00	1.17 ± 0.40 Aab	0.17 ± 0.17 Ba	0.83 ± 0.30	0.33 ± 0.33
2-way ANOVA	Groundcover: $F_{1,50}$=2.81, p= 0.0996; Date: $F_{4,50}$=2.38, p=0.0636; Groundcover*Date: $F_{4,50}$=1.81, p=0.1415		**Groundcover: $F_{1,50}$=20.69, p=0.0001; Date: $F_{4,50}$=7.31, p=0.0001; Groundcover*Date: $F_{4,50}$=7.28, p=0.0001**		Groundcover: $F_{1,50}$=0.01, p=0.906; Date: $F_{4,50}$=2.20, p=0.0819; Groundcover*Date: $F_{4,50}$=0.26, p=0.9018	

Capital letters indicate significant differences between treatments while small letters indicate significant differences between assessment dates. Bold fonts indicate significant statistical differences in 2 way-ANOVA.

IPM: IPM orchard, OP: organic palmette orchard, OG: organic goblet orchard.
